# Human campylobacteriosis: A public health concern of global importance

**DOI:** 10.1016/j.heliyon.2019.e02814

**Published:** 2019-11-14

**Authors:** Aboi Igwaran, Anthony Ifeanyi Okoh

**Affiliations:** a SAMRC Microbial Water Quality Monitoring Centre, University of Fort Hare, Alice, 5700, South Africa; b Applied and Environmental Microbiology Research Group (AEMREG), Department of Biochemistry and Microbiology, University of Fort Hare, Private Bag X1314, Alice, 5700, Eastern Cape, South Africa

**Keywords:** *Campylobacter*, Gastrointestinal, Pathogenesis, Infection, Toxins, Resistance, Microbiology

## Abstract

*Campylobacter* species are among the leading cause of bacterial foodborne and waterborne infections. In addition, *Campylobacter* is one of the major causative agent of bacterial gastrointestinal infections and the rise in the incidence of *Campylobacter* infections have been reported worldwide. Also, the emergence of some *Campylobacter* species as one of the main causative agent of diarrhea and the propensity of these bacteria species to resist the actions of antimicrobial agents; position them as a serious threat to the public health. This paper reviews *Campylobacter* pathogenicity, infections, isolation and diagnosis, their reservoirs, transmission pathways, epidemiology of *Campylobacter* outbreaks, prevention and treatment option, antibiotics resistance and control of antibiotics use.

## 1 Introduction

*Campylobacter* belong to a distinct group of specialized bacteria designated rRNA superfamily VI of Class Proteobacteria (Allos, 2011). *Campylobacter* species are slender Gram-negative rod-shaped, spiral-shaped with single or pair of flagella. Some *Campylobacter* species have multiple flagella such as *C*. *showae* while some species are non-motile like *C*. *gracilis* (Acke, 2018). *Campylobacter* species are indole negative, oxidase positive, hippurate positive, catalase positive, nitrate positive and glucose utilization negative (Pal, 2017). *Campylobacter* species are closely related group of bacteria that principally colonise the gastrointestinal tracts of different animals (El-Gendy et al., 2013). *Campylobacter* species are enormous significance due to the increase in number of species implicated in animals and human's infections (Jamshidi et al., 2008; Kaakoush et al., 2015). Since its first identification, the number of pathogenic *Campylobacter* species that causes animal and human infections are largely classified through phylogenetic means with few as 500–800 bacteria ingestion dose resulting to human disease (Frirdich et al., 2017; Kaakoush et al., 2009). Nonetheless, report has shown that *Campylobacter* doses of 100 cells or less have been linked with human infections (Tribble et al., 2010). The major infection caused by *Campylobacter* is mainly acute diarrhea (Allos et al., 2013; Blaser, 2008) and since 1977, *Campylobacter* species have been known as the major causative agent of acute diarrhea (Skirrow, 1977). *Campylobacter* species have also been reported to be implicated in various human systemic infections including septic thrombophlebitis, endocarditis, neonatal sepsis, pneumonia (Alnimr, 2014), bloodstream infections (BSIs) (Morishita et al., 2013), acute colitis of inflammatory bowel disease and acute appendicitis (Lagler et al., 2016). Other major post-infections that significantly add to *Campylobacter* disease burden include severe demyelinating neuropathy, Guillain-Barré syndrome (GBS) (Scallan et al., 2015), sequelae and Miller-Fisher syndrome (MFS) (Skarp et al., 2016). *Campylobacter* species are also associated with series of gastrointestinal infections like colorectal cancer and Barrett's esophagus (Man, 2011). In small group of patients, *Campylobacter* species have also been reported to be associated with extragastrointestinal infections such as brain abscesses, meningitis, lung infections, bacteremia and reactive arthritis (Man, 2011).

*Campylobacter* is a significant zoonotic causes of bacterial food-borne infection (Hsieh and Sulaiman, 2018) and farm animals are the major reservoir of *Campylobacter* species and the major cause of campylobacteriosis (Grant et al., 2018). Worldwide, farm animals are also the major cause of both bacteria food poisoning (Del Collo et al., 2017) and *Campylobacter* foodborne gastrointestinal infections (Seguino et al., 2018). *Campylobacter* foodborne infection is a problem and an economic burden to human population which caused about 8.4% of the global diarrhea cases (Connerton and Connerton, 2017). *Campylobacter* foodborne infection is a global concern because of the emerging *Campylobacter* species involved in both human infections and *Campylobacter* foodborne outbreaks (CDC, 2014). *Campylobacter* foodborne outbreak is defined as *Campylobacter* infection that involve more than two or more persons as a result of consumption of *Campylobacter* contaminated foods (Mungai et al., 2015). Majority of campylobacteriosis cases are not recognized as outbreaks rather as sporadic episode involving a single family group (Del Collo et al., 2017). Campylobacteriosis is a collective name of infections caused by pathogenic *Campylobacter* species and is characterized by fever, vomiting, watery or bloody diarrhea (Scallan et al*.*, 2015). In general, *Campylobacter* infections are predominantly common in certain age group such as children (below 4) and the aged (above 75) (Lévesque et al., 2013). Other group of people at high risk of *Campylobacter* infections are immunocompromised individuals, hemoglobinopathies patients and those suffering from inflammatory bowel disease (Kennedy et al., 2004). In addition, the risks of *Campylobacter* infections are higher in high income nations than in low income nations (Platts-Mills and Kosek, 2014). In low income nations, a number of environmental sources pose a high risks of human *Campylobacter* infections (Lee et al., 2013); and most outbreaks are caused by consumption of poultry meats and poultry products (Taylor et al., 2013). Poultry meats include meats from laying hens, turkeys, ostriches, ducks and broilers (Epps et al., 2013), and poultry meats and it product cause about 60–80% of the global campylobacteriosis cases (EFSA, 2015).

## 2 Main text

### 2.1 *Campylobacter* species

*Campylobacter* species are divided into Lior serotypes and penner serotypes and over 100 Lior serotypes and 600 penner serotypes have been reported. Among these Lior serotypes and penner serotypes, only the thermotolerant *Campylobacter* species have been reported to have clinical significance (Garcia and Heredia, 2013).

#### 2.1.1 Pathogenic *Campylobacter* species

Worldwide, pathogenic *Campylobacter* species are responsible for the cause of over 400–500 million infections cases each year. Pathogenic *Campylobacter* species known to be implicated in human infections includes *C. jejuni*, *C*. *concisus*, *C*. *rectus*, *C*. *hyointestinalis*, *C*. *insulaenigrae*, *C*. *sputorum*, *C*. *helveticus*, *C*. *lari*, *C*. *fetus*, *C*. *mucosalis*, *C*. *coli*, *C*. *upsaliensis* and *C. ureolyticus* (Heredia and García, 2018). These pathogenic *Campylobacter* species are grouped into major human enteric pathogens (*C. jejuni*, *C. jejuni* subsp. *jejuni (Cjj)*, *C. jejuni* subsp. *doyley* (*Cjd*), *C. coli* and *C. fetus*); minor pathogens (*C. concisus*, *C. upsaliensis, C. lari* and *C. hyointestinalis*) and major veterinary pathogens (*C. fetus* subsp. *venerealis* (*Cfv*) and *C. fetus* subsp. *fetus* (*Cff*)) (Rollins and Joseph, 2000).

#### 2.1.2 *C. jejuni*

*C. jejuni* is a motile, microaerophilic, zoonotic, thermophilic bacterial considered as the leading cause of worldwide foodborne bacterial gastroenteritis (Taheri et al., 2019). It's a member of the genus *Campylobacter* with polar flagella and helical morphology that is used for movement through viscous solutions including the mucus layer of the gastrointestinal tract (Lertsethtakarn et al., 2011). *C. jejuni* is the major enteric pathogen that displays significant strain-to-strain dissimilarities in their pathogenicity patterns (Hofreuter et al., 2006). *C. jejuni* is the major species that caused infections than other pathogenic *Campylobacter* species (Liu et al., 2017) and also the major *Campylobacter* species that regularly cause diarrhea in human (Epps et al., 2013). Infections caused by *C. jejuni* can develop into diverse severities such as mild and self-limiting diarrhea to hemorrhagic colitis and sometimes to meningitis and bacteremia (Burnham and Hendrixson, 2018; Dasti et al., 2010). *C. jejuni* infections are also associated with many secondary complications such as autoimmune neuropathy (Liu et al., 2018), and inflammatory bowel disease (IBD) (Drenthen et al., 2011; Loshaj-Shala et al., 2015). *C. jejuni* is the major *Campylobacter* species that cause disease in young people (Haddock et al., 2010). *C. jejuni* infections can occur via various routes such as through direct contact with companion and farm animals or through waterborne or foodborne transmission (Domingues et al., 2012). *C. jejuni* is a commensal bacterial of chickens which inhabit the chicken intestines at a level >106–108 CFU/g of chicken faeces (Oh et al., 2018) and chickens are the main vector for human campylobacteriosis (Hartley-Tassell et al., 2018). *C. jejuni* consist of two subspecies; *C. jejuni* subsp. *jejuni* (*Cjj*) and *C. jejuni* subsp. *doyley* (*Cjd*) (Man, 2011). The main phenotypic feature generally used to differentiate *Cjj* from *Cjd* strain is the inability of *C. jejuni* subsp. *doyley* to reduce nitrate and also, *Cjd* is also associated with high susceptibility to cephalothin. Clinically, *Cjd* strain causes both enteritis and gastritis (Parker et al., 2007). *C. jejuni* subsp. *jejuni* (*Cjj*) is the main bacterial cause of enteroinvasive diarrhea (Pacanowski et al., 2008) and the major symptoms of *C*. *jejuni* infections include severe enteritis, severe abdominal cramps, fever and bloody diarrhea with mucus (Biswas et al., 2011). In addition, *C. jejuni* has also been reported to be associated with immunoreactive complications like Miller-Fisher syndromes (Dingle et al., 2001).

#### 2.1.3 *C. coli*

*Campylobacter coli* is an S-shaped curved cell measuring about 0.2–0.5 micrometers long with a single flagellum. It's very similar to *C. jejuni*; and both bacteria cause inflammation of the intestine and diarrhea in humans (Prescott et al., 2005). *C. coli* is the second most regularly reported *Campylobacter* species that causes human infections (Crim et al., 2015). *C*. *coli* is grouped into 3 clades (clade 1, 2 and 3). *C*. *coli* clade 1 includes most *C*. *coli* isolated from humans and farm animals. *C*. *coli* clade 1 causes most of human infections whereas infections cause by *C*. *coli* clade 2 and 3 are rare (Johansson et al., 2018). In high income countries, report has shown that *C. coli* is the second most regular cause of campylobacteriosis (Beier et al., 2018). Also in high income countries, *C. coli* infections are usually sporadic and it show seasonal drifts with majority of the infections occurring in early fall or late summer (Allos and Blaser, 2009). The clinical manifestations of *C. coli* infections include watery diarrhea, abdominal pain, vomiting, fever, inflammatory enterocolitis, malaise and nausea (Fitzgerald and Nachamkin, 2007).

#### 2.1.4 *C. fetus*

*C. fetus* is a curved cell, fastidious motile bacterial that majorly cause septic abortion in farm animals. *C. fetus* can cause infection in human and its infection can be acquired through direct contact with animals, through consumption of undercooked contaminated meat or through ingesting food or water contaminated by animal faeces (Koneman et al., 1997). *C. fetus* is grouped into 3 subsp. which includes: *C. fetus* subsp. *venerealis* (*Cfv*), *C. fetus* subsp. *testudinum* (*Cft*) and *C. fetus* subsp. *fetus* (*Cff*) (Iraola et al., 2017). *Cfv* and *Cff* are associated with farm animal infections (Wagenaar et al., 2014); while *Cft* has also been reported to be associated with human infection such as bacteremia (Fitzgerald et al., 2014). *Cff* and *Cfv* are categorized on the basis of their clinical manifestations and mechanisms of transmission (Iraola et al., 2016). *Cff* caused abortion in infected sheep and cattle (On, 2013) and it's an opportunistic human pathogen that largely infects immunecompromised patients (Wagenaar et al., 2014). *Cfv* is reported to be cattle-restricted pathogen (Mshelia et al., 2010), but this species has been isolated from humans and most human infection caused by *C. fetus* strain is majorly caused by *Cff* (Patrick et al., 2013). Some of the major reported symptoms of *C. fetus* infections include endocarditis, meningitis, septicemia, septic arthritis, peritonitis and cellulitis (Hur et al., 2018). *C. fetus* is sometimes responsible for human systemic infections like bloodstream infection in immunosuppressed and immunocompromised individuals (Morishita et al., 2013), but infections are rare (Kienesberger et al., 2014).

#### 2.1.5 *C. lari*

*Campylobacter lari* was previously called *Campylobacter laridis* and is part of the thermotolerant *Campylobacter* species. *C. lari* is grouped into a genotypically and phenotypically diverse *Campylobacter* group that encompasses of the nalidixic-acid susceptible (NASC) group, nalidixic acid-resistant thermophilic *Campylobacter*, the urease-positive thermophilic *Campylobacter* and the urease-producing NASC. These aforementioned groups are all identified as variants of *C. lari* group (Duim et al., 2004). This *C. lari* group is made up of five *Campylobacter* species (*C. subantarcticus*, *C. insulaenigrae*, *C. volucris*, *C. lari* and *C. peloridis*) with other group of strains called UPTC and *C*. *lari*-like strains (Miller et al., 2014). Though, some of these strains formally identified as *C. lari* group were later classified as novel taxa such as *C. volucris* (Debruyne et al., 2010) and *C. peloridis* (Debruyne et al., 2009). *C. lari* is a species within the genus *Campylobacter* and is grouped into two novel subsp. namely; *C*. *lari* subspe. *concheus* (*Clc*) and *C. lari* subsp. *lari* (*Cll*) (Miller et al., 2014). In 1984, *C. lari* was first reported in immunocompromised patient and since then sporadic cases including water-borne *C. lari* outbreaks have been reported (Martinot et al., 2001). *C. lari* has also been reported to be associated with enteritis, purulent pleurisy, bacteremia, urinary tract infection (Werno et al., 2002), reactive arthritis and prosthetic joint infection (Duim et al., 2004).

#### 2.1.6 *C. upsaliensis*

*Campylobacter upsaliensis* is among the thermotolerant *Campylobacter* species and is mostly found in dogs and cats, regardless of whether the animal is sick or healthy (Jaime et al., 2002). *C. upsaliensis* is the third most *Campylobacter* species after *C. jejuni* and *C. coli*. *C. upsaliensis* was named after the city it was first described and thereafter, reports have emerged globally associating this species as a human bacterial enteropathogen (Bourke et al., 1998). *C. upsaliensis* is a well-known *Campylobacter* species that cause diarrhea in felines and canines (Steinhauserova et al., 2000). *C. upsaliensis* is well recognized as a clinically important emerging diarrhea pathogen in both pediatric and immunocompromised persons (Couturier et al., 2012). It is one of the emerging *Campylobacter* species that is associated with human infections including Crohn's disease, neonatal infection, bacteremia, abscesses, meningitis and abortion (Wilkinson et al., 2018). *C. upsaliensis* has also been reported to cause acute or chronic diarrhea in human and diarrhea in dogs (Cecil et al., 2012) though genetic studies have shown that *Campylobacter* strains isolated from dogs and human strains are different (Damborg et al., 2008). In many nations, *C. upsaliensis* is the second reported *Campylobacter* species that cause infections in human after *C. jejuni* (Premarathne et al., 2017).

#### 2.1.7 Other pathogenic *Campylobacter* species

Other pathogenic *Campylobacter* species implicated in human infections includes *C. mucosalis*, *C. curvus*, *C. insulaenigrae*, *C. doylei*, *C. concisus*, *C. helveticus* and *C. rectus* (Cecil et al., 2012). Beside the well-known pathogenic species, other emerging species such as *C*. *sputorum biovar sputorum*, *C*. *gracilis*, *C*. *ureolyticus*, *C*. *peloridis* and *C*. *showae*, have also been reported to be implicated in causing human infections with some life-threatening complications in hospitalized patients (Nishiguchi et al., 2017). Some of these emerging *Campylobacter* species have also been isolated and detected in samples from the axillary nerve, soft tissue lesions, hepatic, lung, bone infections, the cerebrospinal, peritoneal fluid, genitalia, brain abscesses and thoracic empyema of hospitalized patients (Magana et al., 2017). In addition to these life-threatening complications caused by these emerging pathogens, there is a huge gap in tracing the connection between infection and source of human infection (Man, 2011). Furthermore, even with the global incidence of *Campylobacter* species in causing infections, the knowledge of the epidemiology and pathogenesis are still incomplete (Nielsen et al., 2006).

## 3 Pathogenicity of *Campylobacter* species

*Campylobacter* species are of economic importance as they constantly cause foodborne infections due to diverse genes involved in its pathogenicity (Bolton, 2015). *Campylobacter* pathogenicity is based on the virulence factors (Larson et al., 2008) and these virulence factors are multi-factorial in nature and the ability of these bacteria to survival and resist physiological stress also contributes to its pathogenicity (Casabonne et al., 2016; Ketley, 1995). The various virulence related mechanisms displayed by *Campylobacter* species includes invasive properties, oxidative stress defence, toxin production, iron acquisition and its ability to remain viable but non-culturable state (Bhavsar and Kapadnis, 2006). *Campylobacter* invasion, adherence and colonization also add to the pathogenicity of these groups of bacteria (Backert et al., 2013). Other virulence factors of *Campylobacter* include; secretion of some sets of proteins, translocation capabilities and flagella-mediated motility (Biswas et al., 2011).

### 3.1 Motility and flagella

Motility is important for *Campylobacter* survival under diverse chemotactic conditions it comes across in the gastrointestinal tract (Jagannathan and Penn, 2005). In some *Campylobacter* species, the motility system with the flagella involves a chemosensory system that steers flagella movement depending on the environmental conditions where these bacteria are found. *Campylobacter* chemotaxis and flagellin are the two important virulence factors that help lead these bacteria to its colonization site and also help in invading the host cell (van Vliet and Ketley, 2001). Some of these *Campylobacter* motility virulence factors and their encoding genes are σ^54^ promoter regulates gene (*flaB*) and σ^28^ promoter regulates gene (*flaA*) (Hendrixson, 2006). The *flaA* gene appears to be significant for invasion, colonization of the host epithelial cells and adherence to the host gastrointestinal tracts (Jain et al., 2008). The flagellum is composed of structural extracellular filamentous components and a hook-basal body. The hook-basal body comprises of the following: (a) the surface localized hook, (b) the periplasmic rod and associated ring structures and (c) a base embedded in the cytoplasm and inner membrane of the cell (Lertsethtakarn et al., 2011). The hook-basal body is a complex component that is made up of a number of diverse proteins such as FliO, FlhA, FliG, FlhB, FliP, FliF, FliQ, FliR, FliY, FliM and FliN (Carrillo et al., 2004), FlgI, FlgH, FlgE, FliK, FlgE and FliK (Bolton, 2015). The extracellular filament of the flagella is composed of multimers of the protein including flagellin protein (FlaA and FlaB), FlaA (coded by *flaA* gene), and FlaB (coded by *flaB* gene) which is the minor flagellin protein (Lertsethtakarn et al., 2011).

### 3.2 Chemotaxis

Chemotaxis is a method or system by which motile bacteria sense and move to the direction of more favourable conditions and several pathogenic bacteria uses this practice to invade their hosts (Chang and Miller, 2006). *Campylobacter* chemotaxis virulence factors involve in human infections includes chemotaxis proteins; Che A, B, R, V, W and Z encoded by *cheA*, *cheB*, *cheR*, *cheV*, *cheW* and *cheZ* genes (Hamer et al., 2010), Methyl-accepting chemotaxis proteins encoded by *tlp4*, *tlp* and *tlp1* genes (Marchant et al., 2002), the CheY response regulator that is responsible for controlling flagella rotation encoded by *cheY* gene (Hermans et al., 2011) and *Campylobacter* energy taxis system proteins CetB (Aer2) and CetA (Tlp9) encoded by *cetB* and *cetA* gene (Golden and Acheson, 2002).

### 3.3 Adhesion

*Campylobacter* adherence to epithelial cells of the host gastrointestinal tract is a precondition for its colonisation mediated by some adhesins on the bacterial surface (Jin et al., 2001). *Campylobacter* adhesion virulence factors includes outer membrane protein encoded by *cadF* gene, *Campylobacte*r adhesion protein A encoded by *capA* gene, phospholipase A encoded by *pldA* gene, lipoprotein encoded by *jlpA* gene, periplasmic binding protein encoded by *peb1A* gene, fibronectin-like protein A encoded by *flpA* and Type IV secretion system encoded by *virB11* gene (Bolton, 2015). *Campylobacter* adhering to fibronectin F is another important *Campylobacter* virulence factor that enables these bacteria to bind to fibronectin which promotes the bacterium-host cell interactions and colonization (Konkel et al., 2010). Other virulence genes in *Campylobacter* species reported to be linked with human infections responsible for expression of colonization and adherence include *racR*, *dnaJ*, *docA* and *racR* genes (Datta et al., 2003).

### 3.4 Toxin production

*Campylobacter* produce different type of cytotoxins and cytolethal distending toxin (CDT) is one of these toxins (Schulze et al., 1998). CDT is a tripartite toxin that is made up of three subunits encoded by the *cdtA*, *cdtB* and *cdtC* genes. Cytolethal distending toxin activity is determined by these three *cdt* cluster genes (Martinez et al., 2006). These three *cdt* cluster genes are all needed for these toxins to be active (Asakura et al., 2008). The *cdtA* and *C* genes are heterodimeric toxin subunits responsible for toxin binding and internalization of the host cell while *cdtB* is the subunit which encodes for the toxic/active components of the toxin (Abuoun et al., 2005). Cytolethal distending toxins induce diarrhea in both humans and animals by intrusive with the division of cells in the intestinal crypts (Carvalho et al., 2013).

### 3.5 Invasion

Invasion is another virulence mechanism in *Campylobacter* that is carried out by the flagella which also function as an export apparatus in the secretion of non-flagella proteins during host invasion (Poly and Guerry, 2008). There are many virulence genes that are involved in *Campylobacter* invasion mechanism and the products of these genes including flagellin C (*flaC*) and invasion antigens (*cia*) genes. These genes are transported into the host cell's cytoplasm with the aid of flagella secretion system which is vital for invasion and colonisation (Konkel et al., 2004). The secretion of invasion antigens and invasion protein B (*ciaB*) are also important virulence proteins synthesized by *Campylobacter* species which help in the epithelial cells invasion and adhesion of the host gastrointestinal tract (Casabonne et al., 2016). Other important virulence genes and proteins synthesized by *Campylobacter* species including the 73-kDa protein involved in adhesion, the invasion antigen C protein involved in full invasion of INT-407 cells, invasion associated protein gene (*iam*A) implicated in invasion and virulence, the periplasmic protein HtrA responsible for full binding to the epithelial cells, the HtrA chaperone implicated in full folding of out outer membrane protein, the *CiaI* gene implicated in intracellular survival (Bolton, 2015) and *pldA* and *hcp* genes responsible for the expression of invasion (Iglesias-Torrens et al., 2018).

### 3.6 Other virulence mechanism in *Campylobacter* species

Other virulence mechanism that adds to *Campylobacter* pathogenicity is the ability to obtain the necessary nutrient iron needed for its growth from the host body fluids and tissues (van Vliet and Ketley, 2001). Sialyltransferases (*cstII*) activity also add to *Campylobacter* pathogenicity by providing lipooligosaccharide with a defensive barricade that help facilitates in the disruption of the epithelial cells which mimic the action of human ganglioside inducing diarrhea (Pérez-Boto et al., 2010). The *wlaN* gene is implicated in lipopolysaccharide production (Wieczorek et al., 2018). The *spot* gene is responsible for extreme control (Gaynor et al., 2005), the Kat A (catalase) responsible to convert H_2_O_2_ to H_2_O and O_2_ (Bingham-Ramos and Hendrixson, 2008), the *cj0012c* and *cj1371* proteins genes implicated to protect against reactive oxygen species (Garenaux et al., 2008). The Peb4 chaperone is another virulence mechanism in *Campylobacter* that play a significant role in the exporting of proteins to the outer membrane (Kale et al., 2011). Other virulence genes responsible for stress response genes includes the *cosR*, *cj1556*, *spoT*, *ppk1*, *csrA*, *nuoK* and *cprS* and the cell surface modifications genes (*waaF*, *pgp1* and *peb4*) (García-Sánchez et al., 2019). All these aforementioned *Campylobacter* virulence-associated genes have all been reported to be implicated in human infections (Hansson et al., 2018).

## 4 *Campylobacter* infections

**Fig. 1:**
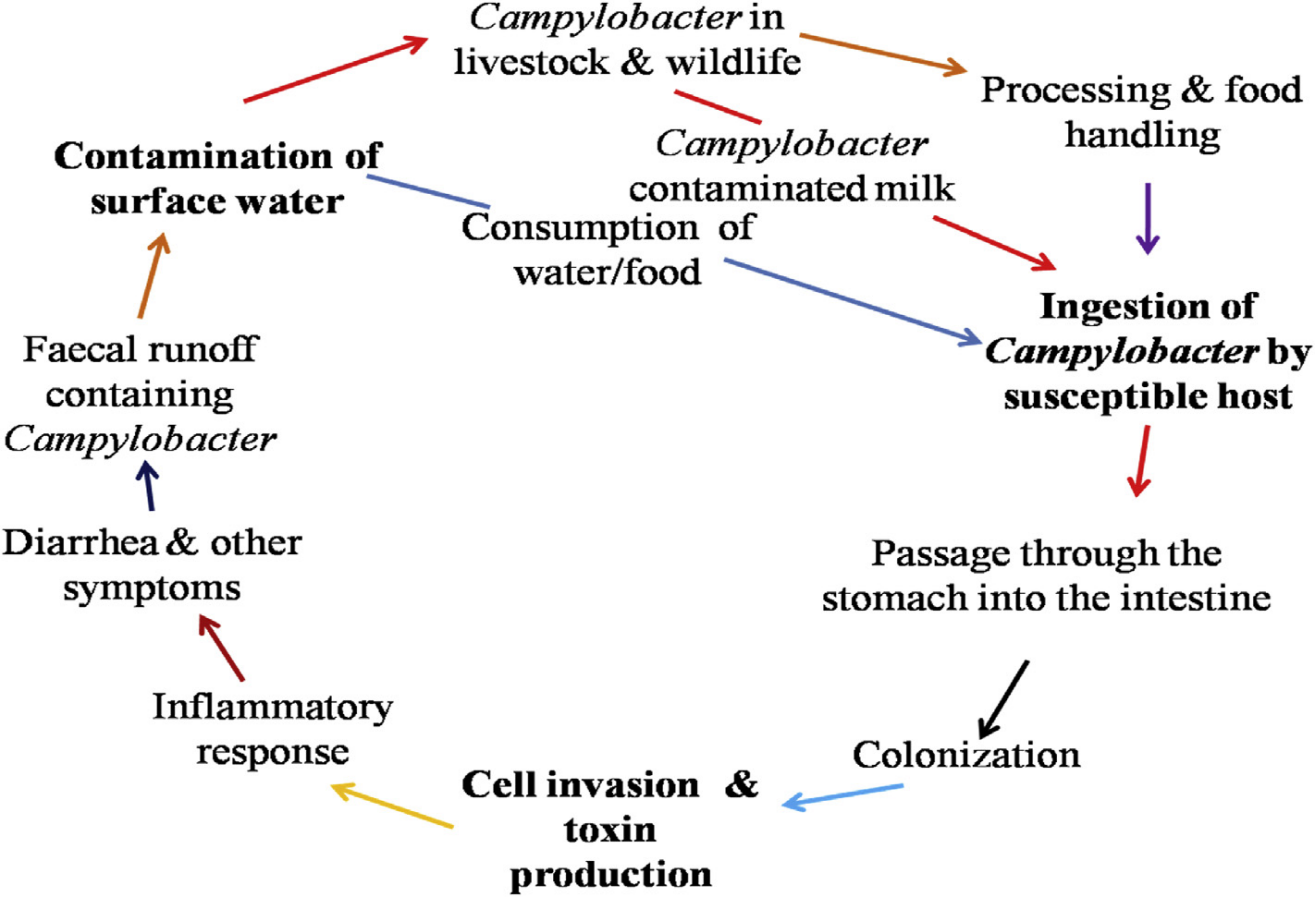
Overview of the transmission cycle involve in *Campylobacter* infections.

*Campylobacters* are types of bacteria that majorly cause infections in the gastrointestinal tract. *Campylobacter* infections may be acquired through different means including consumption of unpasteurized milk, non-chlorinated/contaminated surface water and consumption of undercooked poultry or red meat. *Campylobacter* infections can also be acquired through direct contact with infected pets within the family environment (Shane, 2000). The clinical manifestations of *Campylobacter* infections are oftentimes impossible to differentiate from infections caused by *Shigella* and *Salmonella* (Hansson et al., 2018). *Campylobacter* mechanisms of survival and infection is poorly understood but when colonized the ileum, jejunum and colon, it sometimes causes infection with or without symptoms. Fig. 1 is a schematic representation of the transmission cycle involve in *Campylobacter* infection.

### 4.1 Classification of *Campylobacter* infections

*Campylobacter* infection is a bacterial infection that commonly causes human gastroenteritis but infection can also occur outside the intestines. *Campylobacter* infections are classified into two categories namely; (i): Gastrointestinal infection (GI) and (ii): Extragastrointestinal infection.

#### 4.1.1 Gastrointestinal infections

Gastrointestinal infection (GI) is the inflammation of the gastrointestinal tract involving both the small intestine and stomach (Walsh et al., 2011). GI is generally characterized by diarrhea (Kaakoush et al., 2015). *Campylobacter* is 1 of the 4 key global bacterial cause of gastrointestinal infections (WHO, 2018). It's also the major and regular cause of traveller's diarrhea (Bullman et al., 2011) and children diarrhea (Liu et al., 2016). Besides diarrhea, other gastrointestinal infections associated with different *Campylobacter* species are shown in Table 1.

**Table 1:** *Campylobacter* species associated with human gastroenteritis.

*Campylobacter* species	Gastrointestinal infections
*C. coli*	Gastroenteritis and acute cholecystitis
*C. concisus*	Gastroenteritis and Barrett esophagitis
*C. curvus*	Liver abscess, Barrett esophagitis and gastroenteritis
*C. fetus*	Gastroenteritis
*C. helveticus*	Diarrhea
*C. hominis*	Ulcerative colitis and Crohn's disease
*C. hyointestinalis*	Diarrhea and gastroenteritis
*C. jejuni*	Acute cholecystitis and celiac disease
*C. insulaenigrae*	Abdominal pain, diarrhea and gastroenteritis
*C. lari*	Gastroenteritis and septicaemia
*C. mucosalis*	Gastroenteritis
*C. rectus*	Ulcerative colitis, gastroenteritis and Crohn's disease
*C. showae*	Ulcerative colitis and Crohn's disease
*C. sputorum*	Gastroenteritis
*C. upsaliensis*	Gastroenteritis
*C. ureolyticus*	Gastroenteritis, Crohn's disease and ulcerative colitis

#### 4.1.2 Extragastrointestinal infections

Extragastrointestinal infections (EI) are infections outside the intestines but symptoms are associated with a problem within the intestine (Hernandez and Green, 2006). Extragastrointestinal infections reported to be associated with *Campylobacter* infections includes reactive arthritis, GBS (Kuwabara and Yuki, 2013), bacteremia, septicaemia (Man, 2011), septic arthritis, endocarditis, neonatal sepsis, osteomyelitis, and meningitis (Allos, 2001). In small number of cases, other extragastrointestinal post-infections associated with *Campylobacter* infections include severe neurological dysfunction, neurological disorders and a polio-like form of paralysis (WHO, 2018). Some *Campylobacter* species associated with extragastrointestinal infections are listed in Table 2.

**Table 2:** *Campylobacter* species associated with human extragastrointestinal infections.

*Campylobacter* species	Extragastrointestinal infections
*C. coli*	Bacteremia, sepsis, meningitis and spontaneous abortion
*C. concisus*	Brain abscess, reactive arthritis and rheumatoid arthritis
*C. curvus*	Bronchial abscess and bacteremia
*C. fetus*	Meningitis, vertebral osteomyelitis, brain abscess, cellulitis, septic abortion and bacteremia,
*C. hominis*	Bacteremia
*C. hyointestinalis*	Fatal septicaemia
*C. jejuni*	Sequelae such as bacteremia, urinary tract infection, GBS, reactive arthritis, MFS, sepsis, meningitis and hemolytic uremic syndrome
*C. insulaenigrae*	Septicemia
*C. lari*	Bacteremia
*C. rectus*	Necrotizing soft tissue infection and empyema thoracis
*C. showae*	Intraorbital abscess
*C. sputorum*	Axillary abscess and bacteremia
*C. ureolyticus*	Reactive arthritis and rheumatoid arthritis
*C. upsaliensis*	Breast abscess, bacteremia and spontaneous abortion

### 4.2 Isolation and diognosis of *Campylobacter* infection

Isolation of *Campylobacter* species relied on culture-based methods which have helped to strongly ascertain its part in human infections (Moore et al., 2005). *Campylobacter* isolation involves a medium that uses antibiotics as selective agents. These antibiotics used differs from a single antibiotic including cefoperazone or cefazolin in modified CDA medium to a “cocktail” of polymixin B, trimethroprin and vancomycin found in Skirrow's medium (Thomas, 2005). *Campylobacter* sensitivity to oxidizing radicals and O_2_ has led to the development of a number of selective media and selective agents for its isolation (Silva et al., 2011). Before the development of these culture media for *Campylobacter* isolation and detection, non-selective medium was previously used but the medium was less proper for isolation of campylobacters from environmental and animal samples. Owing to this problem, Bolton and Robertson in 1977 developed a selective Preston medium suitable for *Campylobacter* isolation from environmental and food samples (Bolton and Robertson, 1982). Several other selective broths and media latter developed for *Campylobacter* isolation includes Bolton broth, Preston broth and *Campylobacter* enrichment broth (Baylis et al., 2000), modified charcoal cefoperazone deoxycholate agar (mCCDA) (Wei et al., 2018), CampyFood agar (CFA) and broth, RAPID’*Campylobacter* agar (Seliwiorstow et al., 2014), *Campylobacter* agar base (CAB) and *Campylobacter* Cefex agar (Kashappanavar et al., 2018). *Campylobacter* species are microaerobic, fastidious bacteria capable of growing in a temperature between 37 °C and 42 °C (Davis and DiRita, 2017). Despite *Campylobacter* sensitivity to high temperature and low oxygen concentration, the actual procedures used by clinical laboratories in its isolation from human faecal specimens may vary in different countries (Hurd et al., 2012). However, laboratory diagnosis of campylobacteriosis is usually carried out by culture-base technique or by rapid detection of *Campylobacter* antigen (Enzyme Immunoassay) in stool samples, body tissue or fluids of infected person to identify the genetic materials of this bacterial strain that shows similar symptoms with other bacteria pathogens (Adedayo and Kirkpatrick, 2008; do Nascimento et al., 2016).

Other method use for *Campylobacter* identification includes growth morphology, biochemical tests (Prouzet-Mauléon et al., 2006) and some of these identification methods used are not unreliable (On, 2001). However, other molecular techniques have been designed as alternative and better diagnostic methods for identification (Kuijper et al., 2003). In 1992, application of polymerase chain reaction (PCR) was first used for specific detection of *C. coli* and *C. jejuni* (Oyofo et al., 1992), and PCR is widely used in the detection and identification of this bacterial to species level (Shawky et al., 2015). In addition to PCR techniques, other molecular methods used for identification or detection of *Campylobacter* species include random amplified polymorphic DNA (da Silva et al., 2016), whole-genome sequencing (Hasman et al., 2014; Schürch et al., 2018), matrix-assisted laser desorption/ionization time-of-flight (MALDI-TOF) (Patel, 2019; Singhal et al., 2015). Some of the challenges involved in *Campylobacter* isolation and identification includes difficult procedures for isolation and identification (Llarena et al., 2017), suboptimal storage and loss of isolates during extensive freeze-thaw cycles which has raised concerns to the scientific community (Maziero and de Oliveira, 2010). Likewise, the presence of the “protective guard” in a community of multispecies biofilm could hide a wide range of emerging pathogenic *Campylobacter* species which can successfully “escape” adverse environments and regain its ability to cause infection when found in optimal conditions may lead to wrong results in the diagnosis process (Wood et al., 2013). Another serious challenge for public health concern in campylobacters identification and diagnosis is its ability to remain viable but nonculturable but retain its physiology and virulence ability (Ayrapetyan and Oliver, 2016; Li et al., 2014). Lasstly, the laboratory diagnosis of *Campylobacter* infections caused by other pathogenic *Campylobacter* species except *C. coli* and *C. jejuni* is complex due to the challenging growth and identification processes of the several subsets of *Campylobacter* species (Magana et al., 2017).

### 4.3 Transmission routes of *Campylobacter* infection

**Fig. 2:**
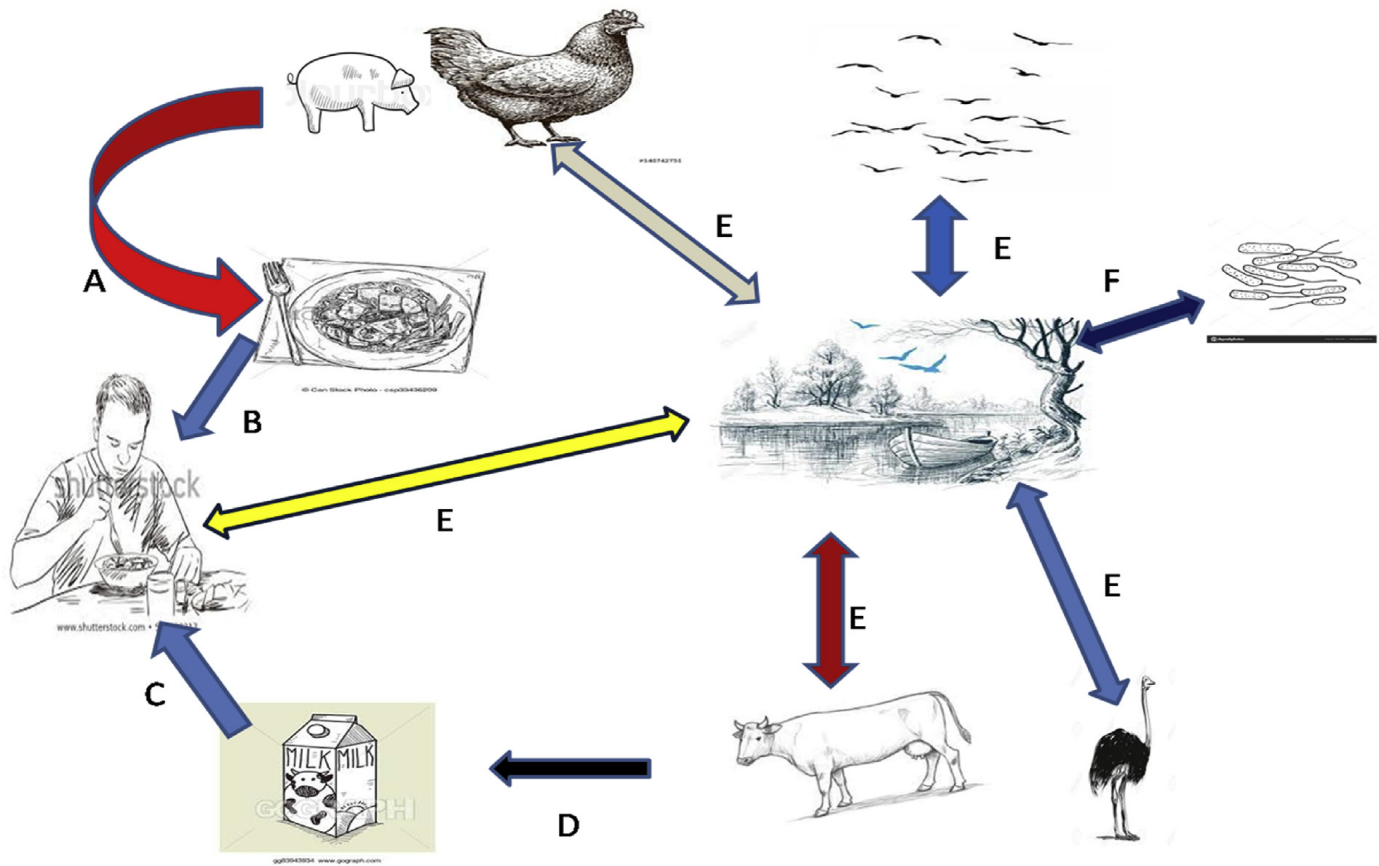
Overview of the transmission routes of *Campylobacter* infection.

*Campylobacter* species majorly colonized the intestine of poultry, European blackbirds, cattle, sheep, ostriches, cats, dogs and pigs (Dearlove et al., 2016). These bacteria are shed in the faeces of these animals into the environment (Goni et al., 2017). *Campylobacter* can also spread to person by direct contact to animals such as pets (ESR, 2016; Westermarck, 2016), with dog owners at high risk of *Campylobacter* infection (Gras et al., 2013). Beside pets, other domestic animals such as cattle are also regularly colonized by *Campylobacter* species and persons working with these animals are also at high risk of *Campylobacter* infection (Hansson et al., 2018). Other sets of people at high risk of campylobacteriosis include farms and abattoirs workers who sometimes do not practice hand-washing and food safety habits (Aung et al., 2015). However, identification and understanding the transmission routes of *Campylobacter* infections is crucial for its prevention and control (Newell et al., 2017). The common and major route/pathways of campylobacteriosis includes through faecal-oral routes (Rosner et al., 2017), through consumption of contaminated undercooked meats or through consumption of contaminated food/water (Grzybowska-Chlebowczyk et al., 2013). Fig. 2 is a schematic illustration of overview of the transmission routes of *Campylobacter* infection.

#### 4.3.1 Milk as a route of *Campylobacter* transmission

Worldwide, there is a rise in the consumption of unpasteurized milk as a result of its health benefits compared to pasteurized milk (Sugrue et al., 2019). Despites the health benefits in the consumption of unpasteurized milk, there is a great concern to the health risk its pose to human (Baars et al., 2019). Milk is a white liquid and a nutrient-rich food produced in the mammary glands of mammals. It's a source of protein, dietary fats and minerals (calcium and magnesium) for growth particularly in children (O'Callaghan et al., 2019). Milk is consumed either unpasteurized or pasteurized and mammals that produced milk for human consumption includes sheep, buffalo, goats, cows, yak and camel and the highest proportions of commercially produced milks are from cows (Quigley et al., 2013). Milk is considered germ-free when secreted in the alveoli of the udder (Vacheyrou et al., 2011). Fresh milk drawn from animals naturally possess a short lived antibacterial system that display ‘germicidal’ or ‘bacteriostatic’ properties but bacterial growth is inevitable after sometimes except it undergoes heat treatment or freezing (Sarkar, 2016). Milk is a good substrate for bacteria growth (Hudson et al., 2014) and it's reported to be among the major transmission routes for *Campylobacter* to humans (El-Zamkan and Hameed, 2016). Milk is natural foods that has no protection against external contamination and can easily be contaminated when separated from it source (Neeta et al., 2014). Milk contamination generally occurs from environmental sources such as water, grass, milking equipments, feed, air, teat apex, soil and other sources (Coorevits et al., 2008). It's believed that the occurrence of *Campylobacter* species in raw milk samples is from faecal contamination (Oliver et al., 2005). *Campylobacter* species have been detected in cow milk (Del Collo et al., 2017 et al., 2017), and different *Campylobacter* species that have been detected in milk samples from different mammals including *C. Jejuni* detected in buffalo and cow milk (Modi et al., 2015) and *C. coli* identified in cow milk (Rahimi et al., 2013). *Campylobacter* species have also been detected in bulk tank milk where these milks are stored (Bianchini et al., 2014), and *Campylobacter* species reported to have been detected from milk samples from the bulk tank include *C. lari*, *C. jejuni* and *C*. *coli* (Del Collo et al., 2017). Globally, several cases of illness and deaths have been reported to occur via consumption of contaminated raw milk and its products (Hati et al., 2018), and in many countries, milk-borne pathogens are of public health concern (Amenu et al., 2019).

#### 4.3.2 Meat as a route of *Camplobacter* transmission

Worldwide, consumption of meats is steadily increasing and meats are sometimes contaminated with microorganisms but bacteria contaminations may sometimes occur from animal microbiota, equipment surfaces and water (Vihavainen et al., 2007). The bacteria from animal's microbiota that majorly contaminate meats include pathogenic *Salmonella* and *Campylobacter* species and these two bacteria species are majorly responsible for human gastroenteritis as a result of consumption of contaminated undercooked meat (Rouger et al., 2017). *Campylobacter* contamination remains the major cause of bacterial food-borne infection and the major reservoir of these bacteria species are poultry (Wieczorek et al., 2015). Infections caused by poultry consumption represents about 50–70% of the global *Campylobacter* infections cases (Seliwiorstow et al., 2015), and poultry is define as meats from chicken, turkey, duck and goose (Szosland-Fałtyn et al., 2018). Beside poultry, *Campylobacter* species have also been detected in other meat typss such as pork and beef (Hussain et al., 2007; Korsak et al., 2015), mutton (Nisar et al., 2018) and in camel, lamb and chevon (Rahimi et al., 2010). *Campylobacter* species that have been isolated and detected in meat samples include *C. coli* and *C. jejuni* identified in poultry meat (Mezher et al., 2016), *C*. *coli*, *C*. *lari*, *C*. *jejuni* and *C*. *fetus* detected in mutton samples (Sharma et al., 2016), and *C*. *jejuni* and *C*. *coli* detected in pork, beef and lamb (Wong et al., 2007). Isolation and detection of these bacteria species from meats samples position them as one of the major transmission route (Duarte et al., 2014).

#### 4.3.3 Water as a route of *Campylobacter* transmission

Worldwide, access to safe drinking water is one of the targets goals, but report from regular analysis of water samples have showed that unsafe drinking water remain the number eight leading risk factor for human disease (Khan and Bakar, 2019). Studies have also showed that improved water sources including public taps/standpipes, protected dug wells, boreholes and protected springs are not automatically free of faecal contamination (Bain et al., 2014). In high income countries, water is sometimes contaminated through faulty pumps and pipes while in low income countries, majority of the people rely mostly on streams, lakes and other surface water sources for food preparation, washing clothes, drinking and these water are usually contaminated by human and animal faeces exacerbating the possibility of waterborne infections (Thompson and Monis, 2012). Though, some environmental sources used for recreational purposes are often overlooked as a route of disease transmission (Henry et al., 2015). Water is an important route of *Campylobacter* transmission to humans resulting to waterborne infections (Mossong et al., 2016) and waterborne infections can involve several persons (Pitkänen, 2013). Besides, *Campylobacter* infection via consumption of contaminated food/tap water (Irena et al., 2008), other water sources including dug well water have been reported to be implicated in *Campylobacter* outbreaks (Guzman-Herrador et al., 2015). Some of the reported pathogenic *Campylobacter* species detected in water samples from beach and river include *C. coli*, *C. jejuni* and *C. lari* (Khan et al., 2013). Other water sources where these bacteria have also been isolated includes ponds, streams, lakes (Sails et al., 2002), children's paddling pool (Gölz et al., 2018), groundwater and seawater (Kemp et al., 2005).

### 4.4 Epidemiological information of *Campylobacter* outbreaks

**Fig. 3:**
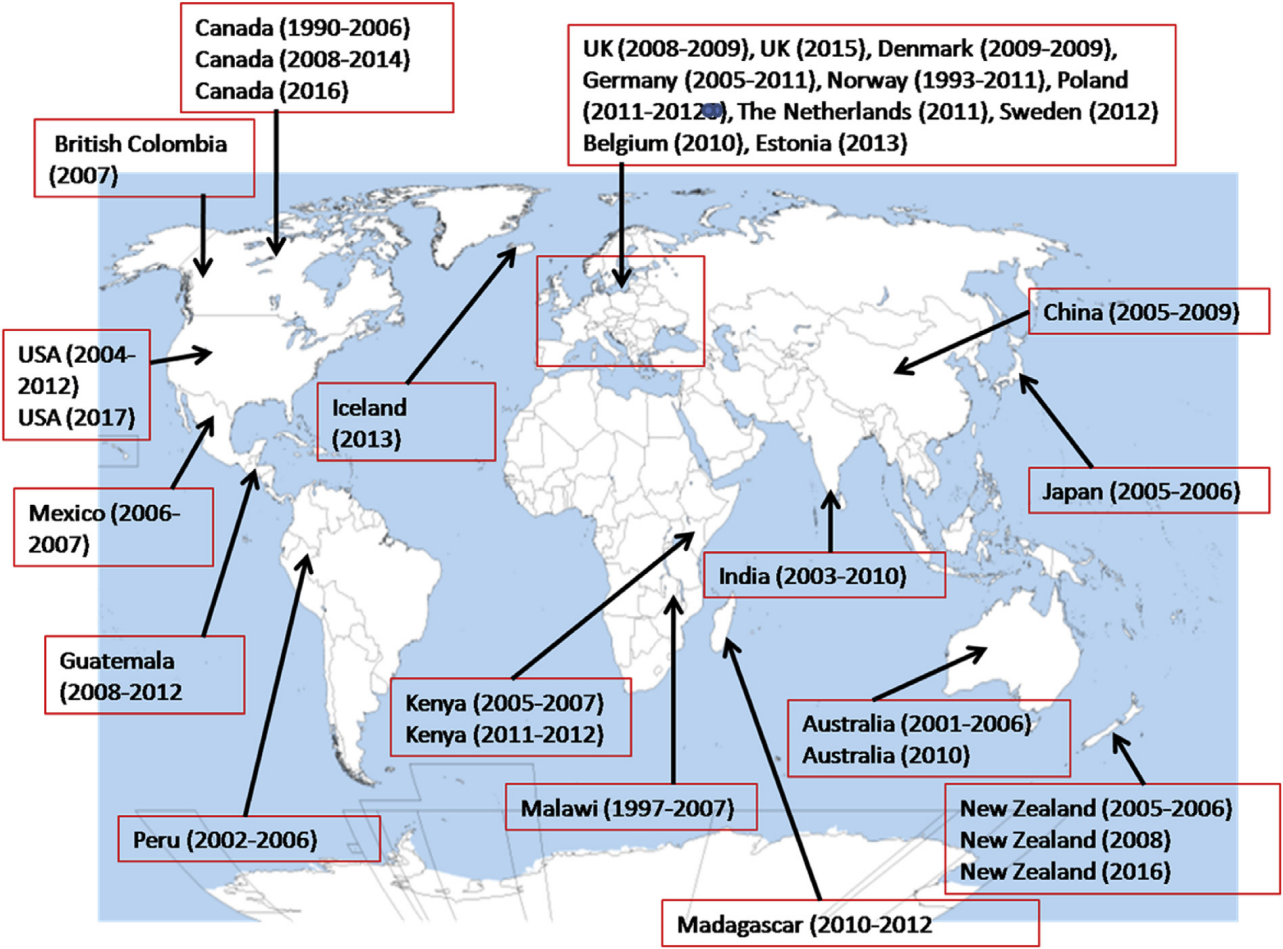
List of some countries with records of campylobacteriosis outbreaks.

The reports in the incidences of *Campylobacter* outbreaks differs among countries and the true nature of the global occurrence rate is largely unknown (WHO, 2013). The reasons for lack of true incidences rate of *Campylobacter* outbreaks includes underreporting of *Campylobacter* infection cases, differences in the reporting systems, difficulties with diagnosis and differences in surveillance in case of outbreaks (Hansson et al., 2018). *Campylobacter* outbreaks are usually either from waterborne or foodborne infection involving several persons (Frost et al., 2002), and majority of *Campylobacter* outbreaks are usually from animal origin (Wilson et al., 2008). Although, in low income countries, *Campylobacter* outbreaks are majorly from environmental sources such as streams and river where many people depend on these water bodies as their major drinking water source (Clark et al., 2003; Platts-Mills and Kosek, 2014). Beside involvement of water sources in human infection in low income countries, water sources have also been reported to be implicated in *Campylobacter* outbreaks in high income countries such as Norway (Jakopanec et al., 2008), New Zealand (Bartholomew et al., 2014), Canada (Clark et al., 2003), Finland (Kuusi et al., 2004) and Denmark (Kuhn et al., 2017). *Campylobacter* milk borne infection and outbreaks have also been reported in several high and low income countries (García-Sánchez et al., 2017). Some of the countries with records of campylobacteriosis outbreaks including the Netherlands (Bouwknegt et al., 2013), Israel (Weinberger et al., 2013), China (Chen et al., 2011), Japan (Kubota et al., 2011), India (Mukherjee et al., 2013), Sweden (Lahti et al., 2017), Mexico (Zaidi et al., 2012) and the United States (Geissler et al., 2017; Gilliss et al., 2013). Also, other nations where there have been records of *Campylobacter* outbreaks includes Canada (Keegan et al., 2009; Ravel et al., 2016), British Columbia (Stuart et al., 2010), Australia (Kaakoush et al., 2015; Unicomb et al., 2009), the United Kingdom (Tam et al., 2012), Belgium (Braeye et al., 2015), Denmark (Nielsen et al., 2013), Germany (Hauri et al., 2013), Norway (Steens et al., 2014), Poland (Sadkowska-Todys and Kucharczyk, 2014), New Zealand (Berger, 2012; Sears et al., 2011), Madagascar (Randremanana et al., 2014), Malawi (Mason et al., 2013), Kenya (O'Reilly et al., 2012; Swierczewski et al., 2013), Iceland and Estonia (Skarp et al., 2016), Guatemala (Benoit et al., 2014) and Peru (Lee et al., 2013). Fig. 3 is a map showing records of some reported cases of *Campylobacter* outbreaks in some countries of the world.

### 4.5 Prevention and treatment of *Campylobacter* infections

Prevention of *Campylobacter* infections can be directly applied to humans by different ways including sewage sanitary conditions, provision of portable water, vaccine usage, public awareness concerning the significance of pasteurization of milk, proper cooking of food from animal origins and the use of therapeutics in case of infections (Hansson et al., 2018). Prevention of *Campylobacter* infections can also be directed on animals by phage treatment (Borie et al., 2014), probiotics, prebiotics, and by improved biosecurity such as the provision of good water quality at farm level and also by monitoring the regular use of antibiotics in animal husbandry. Another vital preventive measure that will help lower the level of these bacteria is the withholding of feed from poultry for about 12 h before slaughter (Hansson et al., 2018). *Campylobacter* infections are sometimes self-limiting but in most cases fluid and electrolyte replacement are major supportive measures for the treatment of this infection (Guarino et al., 2014). Beside fluid and electrolyte replacement, antibiotics are used when symptoms pesist and antibiotics treatments are most effective when started within three days after onset of illness. Nonetheless, antibiotics are regularly used in *Campylobacter* infected patients with diarrhea, high fever or patients with other severe illness like weakened immune systems, AIDS, thalassemia, and hypogammaglobulinemia (CDC, 2016). Antibiotics drugs of choice for the treatment of campylobacteriosis includes fluoroquinolones, aminoglycosides, tetracycline, macrolides, betalactams (Bolton, 2015) and erythromycin (Bardon et al., 2009). Other useful alternative antibiotics drugs of choice include ciprofloxacin, vancomycin (Bruzzese et al., 2018) and quinolones (Gilber and Moellering, 2007).

### 4.6 Antibiotic resistance

**Fig. 4:**
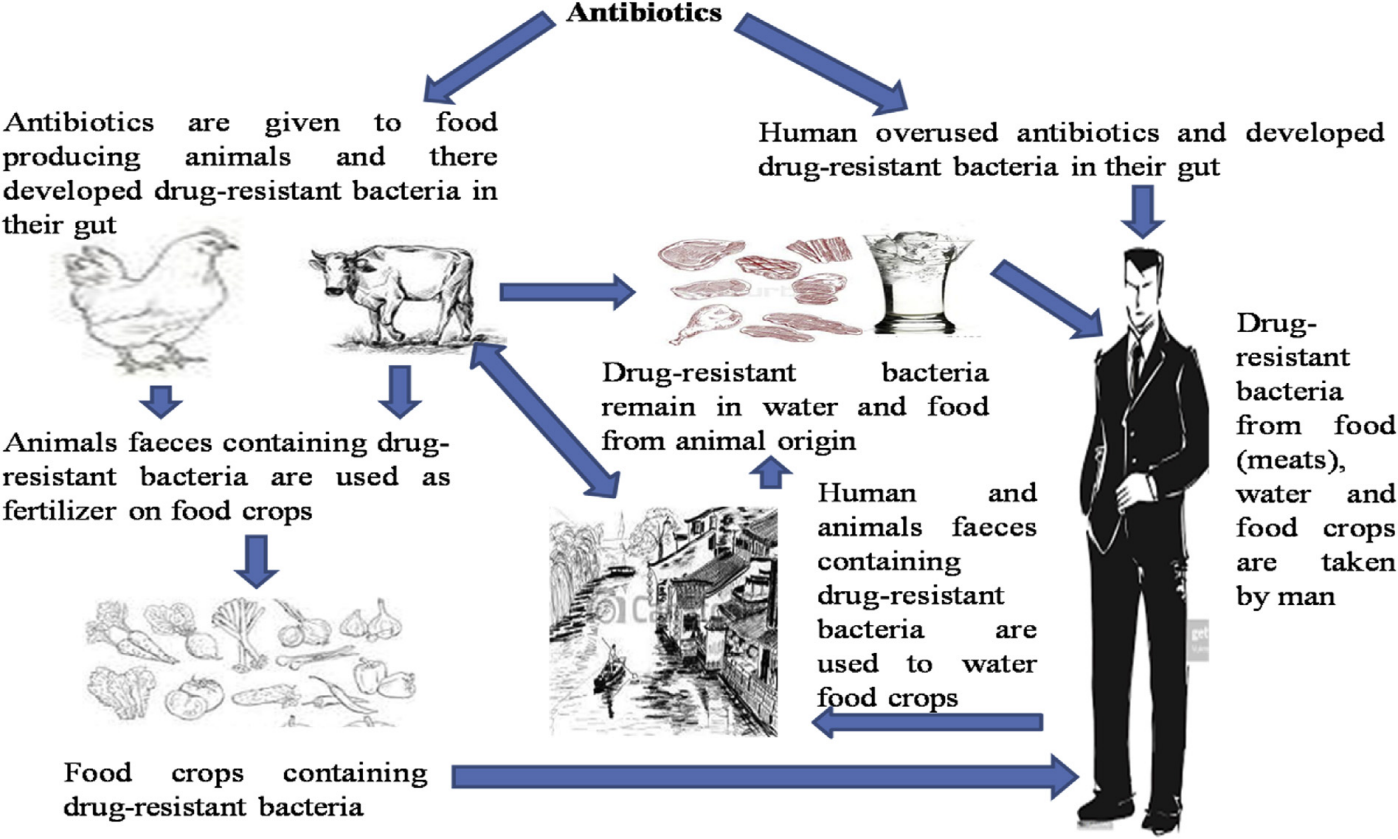
A schematic process involved in the spread of antibiotic resistance bacteria.

Antibiotics use for the treatment of campylobacteriosis is significant for patients with prolonged or severe infections (Reddy and Zishiri, 2017). *Campylobacter* resistance to vital antibiotics used in the treatments of *Campylobacter* infections is an emerging global burden and *Campylobacter* resistance to drugs of choice may limit the treatment options (De Vries et al., 2018). The global spread of antibiotic-resistant *Campylobacter* strains is a contineous process due to the regular use of antibiotics in animal husbandary and this is a problem of public health concern (Silva et al., 2011). Other problems that add to the spread of *Campylobacter* resistance includes inability to completely remove these antibiotic-resistant bacteria during wastewater treatment process, inproper dumping of humans and animals waste into waterbodies and inappropriate preparation of food from animal origin (Founou et al., 2016). Antibiotic resistant bacteria is a global problem assocaited with increased healthcare cost, prolonged infections with a greater risk of hospitalization and high mortality risk and rate (Founou et al., 2017). Molecular detection of antibiotic resistance genes in *Campylobacter* species have helped in determining the resistance genes in *Campylobacter* species from animals and environmental origin (Moyane et al., 2013). Molecular detection of antimicrobial resistance genes in *Campylobacter* originating from foods and water samples is a major public health concern of global importance (Elhadidy et al., 2018). Some of the resistance genes detected in *Campylobacter* species includes quinolone resistance-genes (*gyrA*, *gyrB* and *parC*) (Piddock et al., 2003), FQ-resistant (*parE*) (Luangtongkum et al., 2009), β-lactamase (bla_OXA-61_ and *bla*_OX*-184*_), tetracycline resistance genes (*tet*A, *tet*B, *tet*M, *tet*O and *tet*S) (Reddy and Zishri, 2017), aminoglycoside resistance genes (*aphA* and *aadE*) (García-Sánchez et al., 2019) and erythromycin resistance gene (*erm*B) (Wang et al., 2014). Antibiotics resistance genes in *Campylobacter* are either acquired by spontaneous mutations or through horizontal gene transfer via transduction, conjugation and transformation (Kumar et al., 2016). Other resistance mechanisms developed by *Campylobacter* against antimicrobials include genetic mutation (Reddy and Zishiri, 2017), point mutation (Luangtongkum et al., 2009), decreased in membrane permeability due to MOMP (García-Sánchez et al., 2019) and rRNA methylases (Wang et al., 2014). Resistance-nodulation-cell division efflux system, modification of ribosomal target sites and weakening of the interaction of the macrocyclic ring and the tunnel wall of the ribosome are also essential resistance mechanism developed in *Campylobacter* (Wei, and Kang, 2018). Fig. 4 is a schematic illustration of the patterns implicated in the spread of antibiotic resistance genes.

### 4.7 Control of antibiotic use

The emergence of antibiotic-resistant *Campylobacter* strains has rise markedly in both developing and developed countries suggesting the use of antibiotics in animal husbandry as the source of the accelerating trend (Wieczorek and Osek, 2013). Several countries have policy in the control of antibiotics use in animal production (Maron et al., 2013). However, multiples countries do not have policy in the control of antibiotics use for animal production. In addition, grain-based feeds and water are mostly supplemented with antibiotics and other drugs for animal production (Sapkota et al., 2007). In some countries that practise indiscriminate use of antimicrobial in animal production, new regulatory policy should be place on the use of antibiotic in animal husbandry for non-therapeutic reasons such as promoting weight gains of birds or improving feed efficiency (Rahman et al., 2018). Owing to the increase in antibiotic-resistant *Campylobacter* strains, vaccine development is important and vaccination of birds against *Campylobacter* could help eradicate *Campylobacter* from birds and reduce the rate of incidence of human infections (Avci, 2016). Vaccine would also help to reduce high cost of post-harvest treatments (Johnson et al., 2017). Nevertheless, the cost of *Campylobacter* infections treatment to public health systems is high thus the main motivation towards developing a *Campylobacter* vaccine would be to reduce the high costs of treatment associated with campylobacteriosis, enhance food safety and reduce potential human health risks (Lund and Jensen, 2016). Presently, there are no vaccines approved by any global governing authority to prevent *Campylobacter* infections (Riddle and Guerry, 2016). Vaccine approaches against *Campylobacter* infections are restricted by lacking in comprehension of its association with post-infectious syndromes, antigenic diversity, protective epitopes and its pathogenesis (Riddle and Guerry, 2016).

## 5 Conclusions

Worldwide, outbreaks of campylobacteriosis have been increasing and the major routes of transmission of these bacteria to human is generally believed to be through consumption of contaminated foods. The development of rapid Kits for *Campylobacter* detection and quantification in foods from animal origin will be essential for the prevention of *Campylobacter* infections. *Campylobacter* infections are majorly treated with antibiotics and the actions of these antibiotics have been compromised and this call for the development of new vaccines that will help to control the regular use of antibiotics in animal husbandry. In addition, regular domestic hygiene will also help to prevent *Campylobacter* infections. The production of new and effective antibiotic for better treatment of campylobacteriosis will as well help in the reduction of antibiotic-resistant *Campylobacter* strain and the spread of antibiotics resistant genes.

## Declarations

### Author contribution statement

All authors listed have significantly contributed to the development and the writing of this article.

### Funding statement

This work was supported by the South African Medical Research Council.

### Competing interest statement

The authors declare no conflict of interest.

### Additional information

No additional information is available for this paper.

## References

[bib1] Abuoun M., Manning G., Cawthraw S.A., Ridley A., Ahmed I.H., Wassenaar T.M., Newell D.G. (2005). Cytolethal distending toxin (CDT)-negative *Campylobacter jejuni* strains and anti-CDT neutralizing antibodies are induced during human infection but not during colonization in chickens. Infect. Immun..

[bib2] Adedayo O., Kirkpatrick B.D. (2008). *Campylobacter jejuni* infections: update on presentation, diagnosis, and management. Clin. Rev..

[bib3] Acke E. (2018). Campylobacteriosis in dogs and cats: a review. N. Z. Vet. J..

[bib4] Allos B.M. (2001). *Campylobacter jejuni* infections: update on emerging issues and trends. Clin. Infect. Dis..

[bib5] Allos B.M., Blaser M.J. (2009). Mandell, Douglas, and Bennett's Principles and Practices of Infectious Diseases.

[bib6] Allos B.M. (2011). Microbiology, Pathogenesis, and Epidemiology of *Campylobacter* Infection.

[bib7] Allos B.M., Calderwood S.B., Baron E.L. (2013). Clinical Manifestations, Diagnosis, and Treatment of Campylobacter Infection.

[bib8] Alnimr A.M. (2014). A case of bacteremia caused by *Campylobacter fetus*: an unusual presentation in an infant. Infect. Drug Resist..

[bib9] Amenu K., Wieland B., Szonyi B., Grace D. (2019). Milk handling practices and consumption behavior among Borana pastoralists in southern Ethiopia. J. Health Popul. Nutr..

[bib10] Asakura M., Samosornsuk W., Hinenoya A., Misawa N., Nishimura K., Matsuhisa A., Yamasaki S. (2008). Development of a cytolethal distending toxin (cdt) gene-based species-specific multiplex PCR assay for the detection and identification of *Campylobacter jejuni, Campylobacter coli* and *Campylobacter fetus*. FEMS Immunol. Med. Microbiol..

[bib11] Aung W.W., Saleha A.A., Zunita Z., Murugaiyah M., Aliyu A.B., Goni D.M., Mohamed A.M. (2015). Occurrence of *Campylobacter* in dairy and beef cattle and their farm environment in Malaysia. Pakistan Vet. J..

[bib12] Avci F.Y. (2016). A chicken vaccine to protect humans from diarrheal disease. Glycobiology.

[bib13] Ayrapetyan M., Oliver J.D. (2016). The viable but non-culturable state and its relevance in food safety. Curr. Opin. Food Sci..

[bib14] Baars T., Berge C., Garssen J., Verster J. (2019). The impact of raw milk consumption on gastrointestinal bowel and skin complaints in immune depressed adults. Eur. Neuropsychopharmacol..

[bib15] Backert S., Boehm M., Wessler S., Tegtmeyer N. (2013). Transmigration route of *Campylobacter jejuni* across polarized intestinal epithelial cells: paracellular, transcellular or both?. Cell Commun. Signal..

[bib16] Bain R., Cronk R., Wright J., Yang H., Slaymaker T., Bartram J. (2014). Fecal contamination of drinking-water in low- and middle-income countries: a systematic review and meta-analysis. PLoS Med..

[bib17] Bardon J., Kolar M., Cekanova L., Hejnar P., Koukalova D. (2009). Prevalence of *Campylobacter jejuni* and its resistance to antibiotics in poultry in the Czech Republic. Zoonoses Pub. Health.

[bib18] Bartholomew N., Brunton C., Mitchell P., Williamson J., Gilpin B. (2014). A waterborne outbreak of campylobacteriosis in the South Island of New Zealand due to a failure to implement a multi-barrier approach. J. Water Health.

[bib19] Baylis C.L., MacPhee S., Martin K.W., Humphrey T.J., Betts R.P. (2000). Comparison of three enrichment media for the isolation of *Campylobacter* spp. from foods. J. Appl. Microbiol..

[bib20] Beier R.C., Harvey R.B., Hernandez C.A., Hume M.E., Andrews K., Droleskey R.E., Davidson M.K., Bodeis-Jones S., Young S., Duke S.E., Anderson R.C. (2018). Interactions of organic acids with *Campylobacter coli* from swine. PLoS One.

[bib21] Benoit S.R., Lopez B., Arvelo W., Henao O., Parsons M.B., Reyes L., Moir J.C., Lindblade K. (2014). Burden of laboratory-confirmed *Campylobacter* infections in Guatemala 2008–2012: results from a facility-based surveillance system. J. Epidemiol. Global Health.

[bib22] Berger S.A. (2012). Infectious Diseases of New Zealand. Gideon E-Books 413.

[bib23] Bhavsar S., Kapadnis B. (2006). Virulence factors of *Campylobacter*. Internet J. Microbiol..

[bib24] Bianchini V., Borella L., Benedetti V., Parisi A., Miccolupo A., Santoro E., Recordati C., Luini M. (2014). Prevalence in bulk tank milk and epidemiology of *Campylobacter jejuni* in dairy herds in Northern Italy. Appl. Environ. Microbiol..

[bib25] Bingham-Ramos L.K., Hendrixson D.R. (2008). Characterization of two putative cytochrome peroxidases of *Campylobacter jejuni* involved in promoting commensal colonization of poultry. Infect. Immun..

[bib26] Biswas D., Hannon S.H., Townsend G.G.H., Potter A., Allan B.J. (2011). Genes coding for virulence determinants of *Campylobacter jejuni* in human clinical and cattle isolates from Alberta, Canada, and their potential role in colonization of poultry. Int. Microbiol..

[bib27] Blaser M.J. (2008). Infections due to *Campylobacter* and related species. Principles of Harrison's Internal Med..

[bib28] Bolton F.J., Robertson L. (1982). A selective medium for isolating *Campylobacter jejuni/coli*. J. Clin. Pathol..

[bib29] Bolton D.J. (2015). *Campylobacter* virulence and survival factors. Food Microbiol..

[bib30] Borie C., Robeson J., Galarce N. (2014). Lytic bacteriophages in Veterinary Medicine: a therapeutic option against bacterial pathogens?. Arch. Med. Vet..

[bib31] Bourke B., Chan V.L., Sherman P. (1998). *Campylobacter upsaliensis*: waiting in the wings. Clin. Microbiol. Rev..

[bib32] Bouwknegt M., van Pelt W., Havelaar A.H. (2013). Scoping the impact of changes in population age-structure on the future burden of foodborne disease in The Netherlands, 2020–2060. Int. J. Environ. Res. Public Health.

[bib33] Braeye T., De Schrijver K., Wollants E., Van Ranst M., Verhaegen J. (2015). A large community outbreak of gastroenteritis associated with consumption of drinking water contaminated by river water, Belgium, 2010. Epidemiol. Infect..

[bib34] Bruzzese E., Giannattasio A., Guarino A. (2018). Antibiotic treatment of acute gastroenteritis in children. F1000 Res..

[bib35] Bullman S., Corcoran D., O’Leary J., Lucey B., Byrne D., Sleator R.D. (2011). *Campylobacter ureolyticus*: an emerging gastrointestinal pathogen?. *Campylobacter ureolyticus:* an emerging gastrointestinal pathogen?. FEMS Immunol. Med. Microbiol..

[bib36] Burnham P.M., Hendrixson D.R. (2018). *Campylobacter jejuni*: collective components promoting a successful enteric lifestyle. Nat. Rev. Microbiol..

[bib37] Carrillo C.D., Taboada E., Nash J.H., Lanthier P., Kelly J., Lau P.C., Verhulp R., Mykytczuk O., Sy J., Findlay W.A., Amoako K. (2004). Genome-wide expression analyses of *Campylobacter jejuni* NCTC11168 reveals coordinate regulation of motility and virulence by *flh*A. J. Biol. Chem..

[bib38] Carvalho A.F.D., Silva D.M.D., Azevedo S.S., Piatti R.M., Genovez M.E., Scarcelli E. (2013). Detection of CDT toxin genes in *Campylobacter* spp. strains isolated from broiler carcasses and vegetables in São Paulo, Brazil. Braz. J. Microbiol..

[bib39] Casabonne C., Gonzalez A., Aquili V., Subils T., Balague C. (2016). Prevalence of seven virulence genes of *Campylobacter jejuni* isolated from patients with diarrhea in Rosario, Argentina. Int. J. Infect..

[bib40] Cecil R.L.F., Goldman L., Schafer A.I. (2012). Goldman's cecil medicine, expert consult premium edition--enhanced online features and print.

[bib41] Center for Disease Control and Prevention (2014). Campylobacter.

[bib42] Center for Disease Control and Prevention (2016). Infectious Disease *Campylobacter* Clinical Foodborne Illnesses. http://WWW.cdc.gov.

[bib43] Chang C., Miller J.F. (2006). *Campylobacter jejuni* colonization of mice with limited enteric flora. Infect. Immun..

[bib44] Chen J., Sun X.T., Zeng Z., Yu Y.Y. (2011). *Campylobacter enteritis* in adult patients with acute diarrhea from 2005 to 2009 in Beijing, China. Chin. Med. J..

[bib45] Clark C.G., Price L., Ahmed R., Woodward D.L., Melito P.L., Rodgers F.G., Jamieson F., Ciebin B., Li A., Ellis A. (2003). Characterization of waterborne outbreak–associated *Campylobacter jejuni*, Walkerton, Ontario. Emerg. Infect. Dis..

[bib46] Connerton I.F., Connerton P.L. (2017). *Campylobacter* Foodborne Disease.

[bib47] Coorevits A., De Jonghe V., Vandroemme J., Reekmans R., Heyrman J., Messens W., De Vos P., Heyndrickx M. (2008). Comparative analysis of the diversity of aerobic spore-forming bacteria in raw milk from organic and conventional dairy farms. Syst. Appl. Microbiol..

[bib48] Couturier B.A., Hale D.C., Couturier M.R. (2012). Association of *Campylobacter upsaliensis* with persistent bloody diarrhea. J. Clin. Microbiol..

[bib49] Crim S.M., Griffin P.M., Tauxe R., Marder E.P., Gilliss D., Cronquist A.B., Cartter M., Tobin-D’Angelo M. (2015). Centers for disease control and prevention. Preliminary incidence and trends of infection with pathogens transmitted commonly through food foodborne diseases active surveillance network, 10 U.S. Sites, 2006–2014. MMWR Morb. Mortal. Wkly. Rep..

[bib50] Damborg P., Guardabassi L., Pedersen K., Kokotovic B. (2008). Comparative analysis of human and canine *Campylobacter upsaliensis* isolates by amplified fragment length polymorphism. J. Clin. Microbiol..

[bib51] da Silva D.T., Tejada T.S., Blum-Menezes D., Dias P.A., Timm C.D. (2016). *Campylobacter* species isolated from poultry and humans, and their analysis using PFGE in southern Brazil. Int. J. Food Microbiol..

[bib52] Dasti J.I., Tareen A.M., Lugert R., Zautner A.E., Gross B.U. (2010). *Campylobacter jejuni*; A brief overview on pathogenicity-associated factors and disease mediated mechanisms. Int. J. Med. Microbiol..

[bib53] Datta S., Niwa H., Itoh K. (2003). Prevalence of 11 pathogenic genes of *Campylobacter jejuni* by PCR in strains isolated from humans, poultry meat and broiler and bovine faeces. J. Med. Microbiol..

[bib54] Davis L., DiRita V. (2017). Growth and laboratory maintenance of *Campylobacter jejuni*. Current Protoc. Microbiol..

[bib55] Dearlove B.L., Cody A.J., Pascoe B., Méric G., Wilson D.J., Sheppard S.K. (2016). Rapid host switching in generalist *Campylobacter* strains erodes the signal for tracing human infections. ISME J..

[bib56] Debruyne L., On S.L., De Brandt E., Vandamme P. (2009). Novel *Campylobacter* lari-like bacteria from humans and molluscs: description of *Campylobacter peloridis* sp. nov., *Campylobacter lari* subsp. *concheus* subsp. nov. and *Campylobacter lari* subsp. *lari* subsp. nov. Int. J. Syst. Evol. Microbiol..

[bib57] Debruyne L., Broman T., Bergström S., Olsen B., On S.L., Vandamme P. (2010). *Campylobacter volucris* species nov., isolated from black-headed gulls (Larus ridibundus). Int. J. Syst. Evol. Microbiol..

[bib58] Del Collo L.P., Karns J.S., Biswas D., Lombard J.E., Haley B.J., Kristensen R.C., Kopral C.A., Fossler C.P., Van Kessel J.A.S. (2017). Prevalence, antimicrobial resistance, and molecular characterization of *Campylobacter* spp. in bulk tank milk and milk filters from US dairies. J. Dairy Sci..

[bib59] De Vries S.P., Vurayai M., Holmes M., Gupta S., Bateman M., Goldfarb D., Maskell D.J., Matsheka M.I., Grant A.J. (2018). Phylogenetic analyses and antimicrobial resistance profiles of *Campylobacter* spp. from diarrhoea patients and chickens in Botswana. PLoS One.

[bib60] Dingle K.E., Van Den Braak N., Colles F.M., Price L.J., Woodward D.L., Rodgers F.G., Endtz H.P., Van Belkum A., Maiden M.C.J. (2001). Sequence typing confirms that *Campylobacter jejuni* strains associated with Guillain-Barre and Miller-Fisher syndromes are of diverse genetic lineage, serotype, and flagella type. J. Clin. Microbiol..

[bib61] Domingues A.R., Pires S.M., Halasa T., Hald T. (2012). Source attribution of human campylobacteriosis using a meta-analysis of case-control studies of sporadic infections. Epidemiol. Infect..

[bib62] do Nascimento V.H., da Silva Q.J., Lima I.F.N., Rodrigues T.S., Havt A., Rey L.C., Mota R.M.S., Soares A.M., Singhal M., Weigl B., Guerrant R. (2016). Combination of different methods for detection of *Campylobacter* spp. in young children with moderate to severe diarrhea. J. Microbiol. Methods.

[bib63] Drenthen J., Yuki N., Meulstee J., Maathuis E.M., van Doorn P.A., Visser G.H., Blok J.H., Jacobs B.C. (2011). Guillain–Barré syndrome subtypes related to *Campylobacter* infection. J. Neurol. Neurosurg. Psychiatry.

[bib64] Duarte A., Santos A., Manageiro V., Martins A., Fraqueza M.J., Caniça M., Domingues F.C., Oleastro M. (2014). Human, food and animal *Campylobacter* spp. isolated in Portugal: high genetic diversity and antibiotic resistance rates. Int. J. Antimicrob. Agents.

[bib65] Duim B., Wagenaar J.A., Dijkstra J.R., Goris J., Endtz H.P., Vandamme P.A. (2004). Identification of distinct *Campylobacter lari* genogroups by amplified fragment length polymorphism and protein electrophoretic profiles. Appl. Environ. Microbiol..

[bib66] El-Gendy A.M., Wasfy M.O., Mansour A.M., Oyofo B., Yousry M.M., Klena J.D. (2013). Heterogeneity of *Campylobacter* species isolated from serial stool specimens of Egyptian children using pulsed field gel electrophoresis. Afri. J. Lab. Med..

[bib67] Elhadidy M., Miller W., Arguello H., Álvarez-Ordóñez A., Duarte A., Dierick K., Botteldoorn N. (2018). Genetic basis and clonal population structure of antibiotic resistance in *Campylobacter jejuni* isolated from broiler carcasses in Belgium. Front. Microbiol..

[bib68] El-Zamkan M.A., Hameed K.G.A. (2016). Prevalence of *Campylobacter jejuni* and *Campylobacter coli* in raw milk and some dairy products. Vet. World.

[bib69] Epps S.V., Harvey R.B., Hume M.E., Phillips T.D., Anderson R.C., Nisbet D.J. (2013). Foodborne *Campylobacter*: infections, metabolism, pathogenesis and reservoirs. Int. J. Environ. Res. Public Health.

[bib70] ESR (2016). The Institute of Environmental Science and Research Ltd. Notifiable diseases New Zealand: annual report 2015. Porirua, New Zealand.

[bib71] European Food Safety Authority (EFSA) and ECDC Scientific Report (2015). The European Union summary report on trends and sources of zoonoses, zoonotic agents and food-borne outbreaks in 2013. EFSA J..

[bib72] Fitzgerald C., Nachamkin I., Murray P.R., Baron E.J., Jorgensen J.H., Landry M.L., Pfaller M.A. (2007). *Campylobacter* and arcobacter. Manual of Microbiology.

[bib73] Fitzgerald C., chao Tu Z., Patrick M., Stiles T., Lawson A.J., Santovenia M., Gilbert M.J., Van Bergen M., Joyce K., Pruckler J., Stroika S. (2014). *Campylobacter fetus* subsp. *testudinum* subsp. nov., isolated from humans and reptiles. Int. J. Syst. Evol. Microbiol..

[bib74] Founou L.L., Founou R.C., Essack S.Y. (2016). Antibiotic resistance in the food chain: a developing country-perspective. Front. Microbiol..

[bib75] Frirdich E., Biboy J., Huynh S., Parker C.T., Vollmer W., Gaynor E.C. (2017). Morphology heterogeneity within a *Campylobacter jejuni* helical population: the use of calcofluor white to generate rod-shaped *C. jejuni* 81-176 clones and the genetic determinants responsible for differences in morphology within 11168 strains. Mol. Microbiol..

[bib76] Frost J.A., Gillespie I.A., O’Brien S.J. (2002). Public health implications of *Campylobacter* outbreaks in England and Wales, 1995-1999: epidemiological and microbiological investigations. Epidemiol. Infect..

[bib77] Founou R.C., Founou L.L., Essack S.Y. (2017). Clinical and economic impact of antibiotic resistance in developing countries: a systematic review and meta-analysis. PLoS One.

[bib78] Garenaux A., Jugiau F., Rama F., Jonge R., Denis M., Federighi M., Ritz M. (2008). Survival of *Campylobacter jejuni* strains from different origins under oxidative stress conditions: effect of temperature. Curr. Microbiol..

[bib79] Garcia S., Heredia N.L. (2013). 11 *Campylobacter*. Guide to Foodborne Path.

[bib80] García-Sánchez L., Melero B., Jaime I., Hänninen M.L., Rossi M., Rovira J. (2017). *Campylobacter jejuni* survival in a poultry processing plant environment. Food Microbiol..

[bib81] García-Sánchez L., Melero B., Jaime I., Rossi M., Ortega I., Rovira J. (2019). Biofilm formation, virulence and antimicrobial resistance of different *Campylobacter jejuni* isolates from a poultry slaughterhouse. Food Microbiol..

[bib82] Gaynor E.C., Wells D.H., MacKichan J.K., Falkow S. (2005). The *Campylobacter jejuni* stringent response controls specific stress survival and virulence-associated phenotypes. Mol. Microbiol..

[bib83] Geissler A.L., Bustos C.F., Swanson K., Patrick M.E., Fullerton K.E., Bennett C., Barrett K., Mahon B.E. (2017). Increasing *Campylobacter* infections, outbreaks, and antimicrobial resistance in the United States, 2004–2012. Clin. Infect. Dis..

[bib84] Gilber D.N., Moellering R.C. (2007). The Sanford Guide to Antimicrobial Therapy.

[bib85] Gilliss D., Cronquist A.B., Cartter M., Tobin-D’Angelo M., Blythe D., Smith K., Lathrop S., Zansky S., Cieslak P.R., Dunn J., Holt K.G., Lance S., Crim S.M., Henao O.L., Patrick M., Griffin P.M., Tauxe R.V. (2013). Incidence and trends of infection with pathogens transmitted commonly through food-foodborne diseases active surveillance network, 10 U.S. sites, 1996–2012. MMWR Morb. Mortal. Wkly. Rep..

[bib86] Gölz G., Kittler S., Malakauskas M., Alter T. (2018). Survival of *Campylobacter* in the food chain and the environment. Curr. Clin. Microbiol. Rep..

[bib87] Golden N.J., Acheson D.W. (2002). Identification of motility and autoagglutination *Campylobacter jejuni* mutants by random transposon mutagenesis. Infect. Immun..

[bib88] Goni M.D., Muhammad J., Goje M., Abatcha M.G., Bitrus A.A., Abbas M.A. (2017). *Campylobacter* in dogs and cats; its detection and public health significance: a Review. Adv. Anim. Vet. Sci..

[bib89] Gras L.M., Smid J.H., Wagenaar J.A., Koene M.G.J., Havelaar A.H., Friesema I.H.M., French N.P., Flemming C., Galson J.D., Graziani C., Busani L. (2013). Increased risk for *Campylobacter jejuni* and *C. coli* infection of pet origin in dog owners and evidence for genetic association between strains causing infection in humans and their pets. Epidemiol. Infect..

[bib90] Grant A.J., Maskell D.J., Holmes M.A. (2018). Phylogenetic Analyses and Antimicrobial Resistance Profiles of *Campylobacter* Spp. From Diarrhoea Patients and Chickens in Botswana.

[bib91] Grzybowska-Chlebowczyk U., Kalita B., Flak-Wancerz A., Jasielska M., Więcek S., Wojcieszyn M., Horowska-Ziaja S., Chlebowczyk W., Woś H. (2013). Clinical course of *Campylobacter* infections in children. Pediatr. Pol..

[bib92] Guarino A., Ashkenazi S., Gendrel D., Vecchio A.L., Shamir R., Szajewska H. (2014). European society for pediatric gastroenterology, hepatology, and nutrition/European society for pediatric infectious diseases evidence-based guidelines for the management of acute gastroenteritis in children in Europe. J. Pediatr. Gastroenterol. Nutr..

[bib93] Guzman-Herrador B., Carlander A., Ethelberg S., de Blasio B.F., Kuusi M., Lund V., Löfdahl M., MacDonald E., Nichols G., Schönning C., Sudre B. (2015). Waterborne outbreaks in the Nordic countries, 1998 to 2012. Eurosurveillance.

[bib94] Haddock G., Mullin M., MacCallum A., Sherry A., Tetley L., Watson E., Dagleish M., Smith D.G., Everest P. (2010). *Campylobacter jejuni* 81-176 forms distinct microcolonies on in vitro-infected human small intestinal tissue prior to biofilm formation. Microbiology.

[bib95] Hansson I., Sandberg M., Habib I., Lowman R., Olsson E.E. (2018). Knowledge gaps in control of *Campylobacter* for prevention of campylobacteriosis. Transbound. Emerg. Dis..

[bib96] Hamer R., Chen P.Y., Armitage J.P., Reinert G., Deane C.M. (2010). Deciphering chemotaxis pathways using cross species comparisons. BMC Syst. Biol..

[bib97] Hartley-Tassell L.E., Day C.J., Semchenko E.A., Tram G., Calderon-Gomez L.I., Klipic Z., Barry A.M., Lam A.K., McGuckin M.A., Korolik V. (2018). A peculiar case of *Campylobacter jejuni* attenuated aspartate chemosensory mutant, able to cause pathology and inflammation in avian and murine model animals. Sci. Rep..

[bib98] Hasman H., Saputra D., Sicheritz-Ponten T., Lund O., Svendsen C.A., Frimodt-Møller N., Aarestrup F.M. (2014). Rapid whole-genome sequencing for detection and characterization of microorganisms directly from clinical samples. J. Clin. Microbiol..

[bib99] Hati S., Gawai K., Sreeja V. (2018). Food borne pathogens: a threat to dairy industry. Res. Rev.: J. Dairy Sci. Technol..

[bib100] Hauri A.M., Just M., McFarland S., Schweigmann A., Schlez K., Krahn J. (2013). Campylobacteriosis outbreaks in the state of Hesse, Germany, 2005–2011: raw milk yet again. DMW (Dtsch. Med. Wochenschr.).

[bib101] Henry R., Schang C., Chandrasena G.I., Deletic A., Edmunds M., Jovanovic D., Kolotelo P., Schmidt J., Williamson R., McCarthy D. (2015). Environmental monitoring of waterborne *Campylobacter*: evaluation of the Australian standard and a hybrid extraction-free MPN-PCR method. Front. Microbiol..

[bib102] Heredia N., García S. (2018). Animals as sources of food-borne pathogens: a review. Animal Nutr..

[bib103] Hermans D., Van Deun K., Martel A., Van Immerseel F., Messens W., Heyndrickx M., Haesebrouck F., Pasmans F. (2011). Colonization factors of *Campylobacter jejuni* in the chicken gut. Vet. Res..

[bib104] Hernandez L., Green P. (2006). Extraintestinal manifestations of celiac disease. Curr. Gastroenterol. Rep..

[bib105] Hendrixson D.R. (2006). A phase-variable mechanism controlling the *Campylobacter jejuni* FlgR response regulator influences commensalism. Mol. Microbiol..

[bib106] Hofreuter D., Tsai J., Watson R.O., Novik V., Altman B., Benitez M., Clark C., Perbost C., Jarvie T., Du L., Galán J.E. (2006). Unique features of a highly pathogenic *Campylobacter jejuni* strain. Infect. Immun..

[bib107] Hsieh Y.-H., Sulaiman I.M., Holban A.M., Grumezescu A.M. (2018). Chapter 5 - campylobacteriosis: an emerging infectious foodborne disease. Foodborne Dis.

[bib108] Hudson A., King N., Lake R., Cressey P. (2014). Risk profile: *Campylobacter jejuni/coli* in raw Milk. ESR (Eur. Surg. Res.).

[bib109] Hur K., Lee E., Kang J., Lee Y. (2018). *Campylobacter fetus* peritonitis in a patient with continuous ambulatory peritoneal dialysis: a first case report in Korea. Ann. Clin. Microbiol..

[bib110] Hurd S., Patrick M., Hatch J., Clogher P., Wymore K., Cronquist A.B., Segler S., Robinson T., Hanna S., Fitzgerald G.S.C. (2012). Clinical laboratory practices for the isolation and identification of *Campylobacter* in foodborne diseases active surveillance network (FoodNet) Sites: baseline information for understanding changes in surveillance data. Clin. Infect. Dis..

[bib111] Hussain I., Mahmood M.S., Akhtar M., Khan A. (2007). Prevalence of *Campylobacter* species in meat, milk and other food commodities in Pakistan. Food Microbiol..

[bib112] Iglesias-Torrens Y., Miro E., Guirado P., Llovet T., Muñoz C., Cerdà-Cuéllar M., Madrid C., Balsalobre C., Navarro F. (2018). Population structure, antimicrobial resistance, and virulence-associated genes in *Campylobacter jejuni* isolated from three ecological niches: gastroenteritis patients, broilers, and wild birds. Front. Microbiol..

[bib113] Iraola G., Pérez R., Betancor L., Marandino A., Morsella C., Méndez A., Paolicchi F., Piccirillo A., Tomás G., Velilla A., Calleros L. (2016). A novel real-time PCR assay for quantitative detection of *Campylobacter fetus* based on ribosomal sequences. BMC Vet. Res..

[bib114] Iraola G., Forster S.C., Kumar N., Lehours P., Bekal S., García-Peña F.J., Paolicchi F., Morsella C., Hotzel H., Hsueh P.R., Vidal A. (2017). Distinct *Campylobacter fetus* lineages adapted as livestock pathogens and human pathobionts in the intestinal microbiota. Nat. Commun..

[bib115] Irena J., Katrine B., Line V., Helge L., Tore F., Raisa H. (2008). A Large Waterborne Outbreak of Campylobacteriosis in Norway: the Need to Focus on Distribution System Safety.

[bib116] Jagannathan A., Penn C., Ketley J.M., Konkel M.E. (2005). Motility in *Campylobacter*. Molecular and Cellular Biology.

[bib117] Jain D., Prasad K.N., Sinha S., Husain N. (2008). Differences in virulence attributes between cytolethal distending toxin positive and negative *Campylobacter jejuni* strains. J. Med. Microbiol..

[bib118] Jaime A.L., Joan S., Lee B., Nancy S., Sydney M.H., Eleanor L., Roshan R., Laurene M. (2002). *Campylobacter upsaliensis*: another pathogen for consideration in the United States. Clin. Infect. Dis..

[bib119] Jakopanec I., Borgen K., Vold L., Lund H., Forseth T., Hannula R., Nygård K. (2008). A large waterborne outbreak of campylobacteriosis in Norway: the need to focus on distribution system safety. BMC Infect. Dis..

[bib120] Jamshidi A., Bassami M.R., Farkhondeh T. (2008). Isolation and identification of *Campylobacter* spp. and *Campylobacter coli* from poultry carcasses by conventional culture method and multiplex PCR in Mashhad, Iran. Iran. J. Vet. Res..

[bib121] Jin S., Joe A., Lynett J., Hani E.K., Sherman P., Chan V.L. (2001). *JlpA*, a novel surfaceexposed lipoprotein specific to *Campylobacter jejuni*, mediates adherence to host epithelial cells. Mol. Microbiol..

[bib122] Johnson T.J., Shank J.M., Johnson J.G. (2017). Current and potential treatments for reducing *Campylobacter* colonization in animal hosts and disease in humans. Front. Microbiol..

[bib123] Johansson C., Nilsson A., Kaden R., Rautelin H. (2018). *Campylobacter coli* clade 3 isolates induce rapid cell death in vitro. Appl. Environ. Microbiol..

[bib124] Kaakoush N.O., Baar C., MacKichan J., Schmidt P., Fox E.M., Schuster S.C., Mendz G.L. (2009). Insights into the molecular basis of the microaerophily of three Campylobacterales: a comparative study. Antonie Leeuwenhoek.

[bib125] Kaakoush N.O., Castaño-Rodríguez N., Mitchell H.M., Man S.M. (2015). Global epidemiology of *Campylobacter* infection. Clin. Microbiol. Rev..

[bib126] Kale A., Phansopa C., Suwannachart C., Craven C.J., Rafferty J.B., Kelly D.J. (2011). The virulence factor PEB4 (*CJ0596*) and the periplasmic protein *Cj1289* are two structurally related SurA-like chaperones in the human pathogen *Campylobacter jejuni*. J. Biol. Chem..

[bib127] Kashappanavar S., Nair A., Vanitha H., Mathew B., Baskaran S., Sudheesh L., Menon V. (2018). Comparison of five different enrichment broth-agar combinations for the isolation of *Campylobacter jejuni*. Int. J. Livestock Res..

[bib128] Keegan V.A., Majowicz S.E., Pearl D.L., Marshall B.J., Sittler N., Knowles L., Wilson J.B. (2009). Epidemiology of enteric disease in C-EnterNet’s pilot site-Waterloo region, Ontario, 1990 to 2004. Can. J. Infect Dis. Med. Microbiol..

[bib129] Kemp R., Leatherbarrow A.J.H., Williams N.J., Hart C.A., Clough H.E., Turner J., Wright E.J., French N.P. (2005). Prevalence and genetic diversity of *Campylobacter* spp. in environmental water samples from a 100-square-kilometer predominantly dairy farming area. Appl. Environ. Microbiol..

[bib130] Kennedy M., Villar R., Vugia D.J., Rabatsky-Her T., Farley M.M., Pass M., Smith K., Smith P., Cieslak P.R., Imhoff B., Griffin P.M. (2004). Hospitalizations and deaths due to *Salmonella* infections, FoodNet, 1996-1999. Clin. Infect. Dis..

[bib131] Ketley J.M. (1995). Virulence of *Campylobacter* species: a molecular genetic approach. J. Med. Microbiol..

[bib132] Khan I.U., Hill S., Nowak E., Palmer M.E., Jarjanazi H., Lee D.Y., Mueller M., Schop R., Weir S., Irwin A.M., Winter J. (2013). Investigation of the prevalence of thermophilic *Campylobacter* species at Lake Simcoe recreational beaches. Inland Waters.

[bib133] Khan J.R., Bakar K.S. (2019). Spatial risk distribution and determinants of *E. coli* contamination in household drinking water: a case study of Bangladesh. Int. J. Environ. Health Res..

[bib134] Kienesberger S., Sprenger H., Wolfgruber S., Halwachs B., Thallinger G.G., Perez-Perez G.I., Blaser M.J., Zechner E.L., Gorkiewicz G. (2014). Comparative genome analysis of *Campylobacter fetus* subspecies revealed horizontally acquired genetic elements important for virulence and niche specificity. PLoS One.

[bib135] Koneman E.W., Allen S.D., Janda W.M., Schreckenberger P.C., Winn W.C. (1997). Diagnostic Microbiology. *The Nonfermentative Gram-Negative Bacilli*.

[bib136] Konkel M.E., Klena J.D., Rivera-Amill V., Monteville M.R., Biswas D., Raphael B., Mickelson J. (2004). Secretion of virulence proteins from *Campylobacter jejuni* is dependent on a functional flagellar export apparatus. J. Bacteriol..

[bib137] Konkel M.E., Larson C.L., Flanagan R.C. (2010). *Campylobacter jejuni* FlpA binds fibronectin and is required for maximal host cell adherence. J. Bacteriol..

[bib138] Korsak D., Maćkiw E., Rożynek E., Żyłowska M. (2015). Prevalence of *Campylobacter* species in retail chicken, Turkey, pork, and beef meat in Poland between 2009 and 2013. J. Food Prot..

[bib139] Kubota K., Kasuga F., Iwasaki E., Inagaki S., Sakurai Y., Komatsu M., Toyofuku H., Angulo F.J., Scallan E., Morikawa K. (2011). Estimating the burden of acute gastroenteritis and foodborne illness caused by *Campylobacter, Salmonella*, and *Vibrio parahaemolyticus* by using population based telephone survey data, Miyagi Prefecture, Japan, 2005 to 2006. J. Food Prot..

[bib140] Kuhn K.G., Falkenhorst G., Emborg H.D., Ceper T., Torpdahl M., Krogfelt K.A., Ethelberg S., Mølbak K. (2017). Epidemiological and serological investigation of a waterborne *Campylobacter jejuni* outbreak in a Danish town. Epidemiol. Infect..

[bib141] Kuijper E.J., Stevens S., Imamura T., De Wever B., Claas E.C. (2003). Genotypic identification of erythromycin-resistant *Campylobacter* isolates as *Helicobacter* species and analysis of resistance mechanism. J. Clin. Microbiol..

[bib142] Kumar A., Drozd M., Pina-Mimbela R., Xu X., Helmy Y.A., Antwi J., Fuchs J.R., Nislow C., Templeton J., Blackall P.J., Rajashekara G. (2016). Novel anti-Campylobacter compounds identified using high throughput screening of a pre-selected enriched small molecules library. Front. Microbiol..

[bib143] Kuwabara S., Yuki N. (2013). Axonal Guillain-Barre syndrome: concepts and controversies. Lancet Neurol..

[bib144] Kuusi M., Klemets P., Miettinen I., Laaksonen I., Sarkkinen H., Hänninen M.L., Rautelin H., Kela E., Nuorti J.P. (2004). An outbreak of gastroenteritis from a non-chlorinated community water supply. J. Epidemiol. Community Health.

[bib145] Lagler H., Kiesewetter B., Raderer M. (2016). Infection with multidrug-resistant *Campylobacter coli* mimicking recurrence of carcinoid syndrome: a case report of a neuroendocrine tumor patient with repeated diarrhea. BMC Infect. Dis..

[bib146] Lahti E., Löfdahl M., Ågren J., Hansson I., Olsson E.E. (2017). Confirmation of a campylobacteriosis outbreak associated with chicken liver pâté using PFGE and WGS. Zoonoses Publ. Health.

[bib147] Lund M., Jensen J.D. (2016). A real options approach to biotechnology investment policy-the case of developing a *Campylobacter* vaccine to poultry. Prev. Vet. Med..

[bib148] Larson C.L., Shah D.H., Dhillon A.S. (2008). *Campylobacter jejuni* invade chicken LMH cells inefficiently and stimulate differential expression of the chicken CXCLi1 and CXCLi2 cytokines. Microbiology.

[bib149] Lee G., Pan W., Yori P.P., Olortegui M.P., Tilley D., Gregory M., Oberhelman R., Burga R., Chavez C.B., Kosek M. (2013). Symptomatic and asymptomatic *Campylobacter* infections associated with reduced growth in Peruvian children. PLoS Neglected Trop. Dis..

[bib150] Lertsethtakarn P., Ottemann K.M., Hendrixson D.R. (2011). Motility and chemotaxis in *Campylobacter* and *Helicobacter*. Annu. Rev. Microbiol..

[bib151] Lévesque S., Fournier E., Carrier N., Frost E., Arbeit R.D., Michaud S. (2013). Campylobacteriosis in urban versus rural areas: a case-case study integrated with molecular typing to validate risk factors and to attribute sources of infection. PLoS One.

[bib152] Li L., Mendis N., Trigui H., Oliver J.D., Faucher S.P. (2014). The importance of the viable but non-culturable state in human bacterial pathogens. Front. Microbiol..

[bib153] Liu J., Platts-Mills J.A., Juma J., Kabir F., Nkeze J., Okoi C., Operario D.J., Uddin J., Ahmed S., Alonso P.L., Antonio M. (2016). Use of quantitative molecular diagnostic methods to identify causes of diarrhoea in children: a reanalysis of the GEMS case-control study. The Lancet.

[bib154] Liu K.C., Jinneman K.C., Neal-McKinney J., Wu W.H., Rice D.H. (2017). Simultaneous identification of *Campylobacter jejuni, Campylobacter coli*, and *Campylobacter lari* with smart cycler-based multiplex quantitative polymerase chain reaction. Foodb. Pathog. Dis..

[bib155] Liu F., Ma R., Wang Y., Zhang L. (2018). The clinical importance of *Campylobacter concisus* and other human hosted *Campylobacte*r species. Front. Cell. Infect. Microbiol..

[bib156] Llarena A.K., Taboada E., Rossi M. (2017). Whole-genome sequencing in epidemiology of *Campylobacter jejuni* infections. J. Clin. Microbiol..

[bib157] Loshaj-Shala A., Regazzoni L., Daci A., Orioli M., Brezovska K., Panovska A.P., Beretta G., Suturkova L. (2015). Guillain Barré syndrome (GBS): new insights in the molecular mimicry between *C. jejuni* and human peripheral nerve (HPN) proteins. J. Neuroimmunol..

[bib158] Luangtongkum T., Jeon B., Han J., Plummer P., Logue C.M., Zhang Q. (2009). Antibiotic resistance in *Campylobacter*: emergence, transmission and persistence. Future Microbiol..

[bib159] Magana M., Chatzipanagiotou S., Burriel A.R., Ioannidis A. (2017). Inquiring into the gaps of *Campylobacter* surveillance methods. Vet. Sci..

[bib160] Man S.M. (2011). The clinical importance of emerging *Campylobacter* species. Nat. Rev. Gastroenterol. Hepatol..

[bib161] Marchant J., Wren B., Ketley J. (2002). Exploiting genome sequence: predictions for mechanisms of *Campylobacter* chemotaxis. Trends Microbiol..

[bib162] Maron D.F., Smith T.J., Nachman K.E. (2013). Restrictions on antimicrobial use in food animal production: an international regulatory and economic survey. Glob. Health.

[bib163] Martinot M., Jaulhac B., Moog R., De Martino S., Kehrli P., Monteil H., Piemont Y. (2001). *Campylobacter lari* bacteremia. Clin. Microbiol. Infect..

[bib164] Martinez I., Mateo E., Churruca E., Girbau C., Alonso R., Fernandez-Astorga A. (2006). Detection of *cdtA, cdtB*, and *cdtC* genes in *Campylobacter jejuni* by multiplex PCR. Int. J. Med. Microbiol..

[bib165] Mason J., Iturriza-Gomara M., O’Brien S.J., Ngwira B.M., Dove W., Maiden M.C., Cunliffe N.A. (2013). *Campylobacter* infection in children in Malawi is common and is frequently associated with enteric virus coinfections. PLoS One.

[bib166] Maziero M.T., de Oliveira T.C. (2010). Effect of refrigeration and frozen storage on the *Campylobacter jejuni* recovery from naturally contaminated broiler carcasses. Braz. J. Microbiol..

[bib167] Mezher Z., Saccares S., Marcianò R., De Santis P., Rodas E.M.F., De Angelis V., Condoleo R. (2016). Occurrence of *Campylobacter* spp. in poultry meat at retail and processing plants’ levels in Central Italy. Ital. J. Food Saf..

[bib168] Miller W.G., Yee E., Chapman M.H., Smith T.P., Bono J.L., Huynh S., Parker C.T., Vandamme P., Luong K., Korlach J. (2014). Comparative genomics of the *Campylobacter lari* group. Gen. Biol. Evol..

[bib169] Modi S., Brahmbhatt M.N., Chatur Y.A., Nayak J.B. (2015). Prevalence of *Campylobacter* species in milk and milk products, their virulence gene profile and antibiogram. Vet. World.

[bib170] Moore J.E., Corcoran D., Dooley J.S., Fanning S., Lucey B., Matsuda M., McDowell D.A., Mégraud F., Millar B.C., O’Mahony R., O’Riordan L. (2005). Campylobacter. Vet. Res..

[bib171] Morishita S., Fujiwara H., Murota H., Maeda Y., Hara A., Horii T. (2013). Bloodstream infection caused by *Campylobacter lari*. J. Infect. Chemother..

[bib172] Mossong J., Mughini-Gras L., Penny C., Devaux A., Olinger C., Losch S., Cauchie H.M., Van Pelt W., Ragimbeau C. (2016). Human campylobacteriosis in Luxembourg, 2010-2013: a case-control study combined with Multilocus sequence typing for source attribution and risk factor analysis. Sci. Rep..

[bib173] Moyane J.N., Jideani A.I.O., Aiyegoro O.A. (2013). Antibiotics usage in food producing animals in South Africa and impact on human: antibiotic resistance. Afr. J. Microbiol. Res..

[bib174] Mshelia G.D., Amin J.D., Woldehiwet Z., Murray R.D., Egwu G.O. (2010). Epidemiology of bovine venereal campylobacteriosis: geographic distribution and recent advances in molecular diagnostic techniques. Reprod. Domest. Anim..

[bib175] Mukherjee P., Ramamurthy T., Bhattacharya M.K., Rajendran K., Mukhopadhyay A.K. (2013). *Campylobacter jejuni* in hospitalized patients with diarrhea, Kolkata, India. Emerg. Infect. Dis..

[bib176] Mungai E.A., Behravesh C.B., Gould L.H. (2015). Increased outbreaks associated with nonpasteurized milk, United States, 2007-2012. Emerg. Infect. Dis..

[bib177] Neeta P.N., Prashanth N., Shivaswamy M.S., Mallapur M.D. (2014). A study on awareness regarding milk borne diseases in an urban community of Karnataka. Int. J. Med. Sci. Public Health.

[bib178] Newell D.G., Mughini-Gras L., Kalupahana R.S., Wagenaa J.A. (2017). *Campylobacter* epidemiology-sources and routes of transmission for human infection. Camp. Featu. Detect. Prevent. Foodbor. Dis..

[bib179] Nielsen E.M., Fussing V., Engberg J., Nielsen N.L., Neimann J. (2006). Most *Campylobacter* subtypes from sporadic infections can be found in retail poultry products and food animals. Epidemiol. Infect..

[bib180] Nielsen H.L., Ejlertsen T., Engberg J., Nielsen H. (2013). High incidence of *Campylobacter* concisusin gastroenteritis in North Jutland, Denmark: a population-based study. Clin. Microbiol. Infect..

[bib181] Nisar M., Mushtaq M.H., Shehzad W., Hussain A., Nasar M., Nagaraja K.V., Goyal S.M. (2018). Occurrence of *Campylobacter* in retail meat in Lahore, Pakistan. Acta Trop..

[bib182] Nishiguchi S., Sekine I., Kuroda S., Sato M., Kitagawa I. (2017). Myositis Ossificans of the hip due to pyogenic arthritis caused by *Campylobacter fetus* Subspecies *fetus*. Intern. Med..

[bib183] O’Callaghan T.F., Sugrue I., Hill C., Ross R.P., Stanton C. (2019). Nutritional aspects of raw milk: a beneficial or hazardous food choice. Raw Milk.

[bib184] Oh E., Chui L., Bae J., Li V., Ma A., Mutschall S.K., Taboada E.N., McMullen L.M., Jeon B. (2018). Frequent implication of multistress-tolerant *Campylobacter jejuni* in human infections. Emerg. Infect. Dis..

[bib185] O'Reilly C.E., Jaron P., Ochieng B., Nyaguara A., Tate J.E., Parsons M.B., Bopp C.A., Williams K.A., Vinjé J., Blanton E., Wannemuehler K.A. (2012). Risk factors for death among children less than 5 years old hospitalized with diarrhea in rural western Kenya, 2005-2007: a cohort study. PLoS Med..

[bib186] Oliver S.P., Jayarao B.M., Almeida R.A. (2005). Foodborne pathogens in milk and the dairy farm environment: food safety and public health implications. Foodb. Pathog. Dis..

[bib187] On S.L. (2001). Taxonomy of *Campylobacter, Arcobacter, Helicobacter* and related bacteria: current status, future prospects and immediate concerns. J. Appl. Microbiol..

[bib188] On S.L.W. (2013). Isolation, identification and subtyping of *Campylobacter*: where to from here?. J. Microbiol. Methods.

[bib189] Oyofo B.A., Thornton S.A., Burr D.H., Trust T.J., Pavlovskis O.R., Guerry P. (1992). Specific detection of *Campylobacter jejuni* and *Campylobacter coli* by using polymerase chain reaction. J. Clin. Microbiol..

[bib190] Pacanowski J., Lalande V., Lacombe K., Boudraa C., Lesprit P., Legrand P., Trystram D., Kassis N., Arlet G., Mainardi J.L., Doucet-Populaire F. (2008). *Campylobacter* bacteremia: clinical features and factors associated with fatal outcome. Clin. Infect. Dis..

[bib191] Pal M. (2017). *Campylobacter jejuni*: an emerging foodborne pathogen of global significance. J. Exp. Food Chem..

[bib192] Parker C.T., Miller W.G., Horn S.T., Lastovica A.J. (2007). Common genomic features of *Campylobacter jejuni* subsp. *doylei* strains distinguish them from *C. jejuni* subsp. *jejuni*. BMC Microbiol..

[bib193] Patel R. (2019). A moldy application of MALDI: MALDI-ToF mass spectrometry for fungal identification. J. Fungi..

[bib194] Patrick M.E., Gilbert M.J., Blaser M.J., Tauxe R.V., Wagenaar J.A., Fitzgerald C. (2013). Human infections with new subspecies of *Campylobacter fetus*. Emerg. Infect. Dis..

[bib195] Pérez-Boto D., López-Portolés J., Simón C., Valdezate S., Echeita M. (2010). Study of the molecular mechanisms involved in high-level macrolide resistance of Spanish *Campylobacter jejuni* and *Campylobacter coli* strains. J. Antimic. Chem..

[bib196] Piddock L.J., Ricci V., Pumbwe L., Everett M.J., Griggs D.J. (2003). Fluoroquinolone resistance in *Campylobacter* species from man and animals: detection of mutations in topoisomerase genes. J. Antimic. Chem..

[bib197] Pitkänen T. (2013). Review of *Campylobacter* species in drinking and environmental waters. J. Microbiol. Methods.

[bib198] Platts-Mills J.A., Kosek M. (2014). Update on the burden of *Campylobacter* in developing countries. Curr. Opin. Infect. Dis..

[bib199] Poly F., Guerry P. (2008). Pathogenesis of *Campylobacter*. Curr. Opin. Gastroenterol..

[bib200] Prescott L.M., Harley J.P., Klein D.A. (2005). *Campylobacter*. Microbiology.

[bib201] Premarathne J.M.K.J.K., Satharasinghe D.A., Huat J.T.Y., Basri D.F., Rukayadi Y., Nakaguchi Y., Nishibuchi M., Radu S. (2017). Impact of human *Campylobacter* infections in Southeast Asia: the contribution of the poultry sector. Crit. Rev. Food Sci. Nutr..

[bib202] Prouzet-Mauléon V., Labadi L., Bouges N., Ménard A., Mégraud F. (2006). *Arcobacter butzleri*: underestimated enteropathogen. Emerg. Infect. Dis..

[bib203] Quigley L., O’Sullivan O., Stanton C., Beresford T.P., Ross P.R., Fitzgerald G.F., Paul D.C. (2013). The complex microbiota of raw milk. FEMS Microbiol. Rev..

[bib204] Rahimi E., Ameri M., Kazemeini H.R. (2010). Prevalence and antimicrobial resistance of *Campylobacter* species isolated from raw camel, beef, lamb, and goat meat in Iran. Foodb. Pathog. Dis..

[bib205] Rahimi E., Sepehri S., Momtaz H. (2013). Prevalence of *Campylobacter* species in milk and dairy products in Iran. Revue de Méd. Vétér..

[bib206] Rahman G., Price L., Rabanes H.G., Hilsabeck R. (2018). Implications of Foodborne Bacteria on Human Health: Isolation and Antibiotic Resistance of *Salmonella enterica* and *Campylobater* Spp. On Retail Chicken Sold in California.

[bib207] Randremanana R.V., Randrianirina F., Sabatier P., Rakotonirina H.C., Randriamanantena A., Razanajatovo I.M., Ratovoson R., Richard V. (2014). *Campylobacter* infection in a cohort of rural children in Moramanga, Madagascar. BMC Infect. Dis..

[bib208] Ravel A., Pintar K., Nesbitt A., Pollari F. (2016). Non food-related risk factors of campylobacteriosis in Canada: a matched case-control study. BMC Public Health.

[bib209] Reddy S., Zishiri O.T. (2017). Detection and prevalence of antimicrobial resistance genes in *Campylobacter* spp. isolated from chickens and humans. Onderstepoort J. Vet. Res..

[bib210] Riddle M.S., Guerry P. (2016). Status of vaccine research and development for *Campylobacter jejuni*. Vaccine.

[bib211] Rosner B.M., Schielke A., Didelot X., Kops F., Breidenbach J., Willrich N., Gölz G., Alter T., Stingl K., Josenhans C., Suerbaum S. (2017). A combined case-control and molecular source attribution study of human *Campylobacter* infections in Germany, 2011-2014. Sci. Rep..

[bib212] Rollins D.M., Joseph S.W. (2000). Pathogenic Microbiology-Fall. *Enterococcus* Summary.

[bib213] Rouger A., Tresse O., Zagorec M. (2017). Bacterial contaminants of poultry meat: sources, species, and dynamics. Microorganisms.

[bib214] Sadkowska-Todys M., Kucharczyk B. (2014). Campylobacteriosis in Poland in 2012. Przegl. Epidemiol..

[bib215] Sails A.D., Bolton F.J., Fox A.J., Wareing D.R.A., Greenway D.L.A. (2002). Detection of *Campylobacter jejuni* and *Campylobacter coli* in environmental waters by PCR enzyme-linked immunosorbent assay. Appl. Environ. Microbiol..

[bib216] Sapkota A.R., Lefferts L.Y., McKenzie S., Walker P. (2007). What do we feed to food-production animals? A review of animal feed ingredients and their potential impacts on human health. Environ. Health Perspect..

[bib217] Sarkar S. (2016). Microbiological safety concerns of raw milk. J. Food Nutr. Diet..

[bib218] Scallan E., Hoekstra R.M., Mahon B.E., Jones T.F., Griffin P.M. (2015). An assessment of the human health impact of seven leading foodborne pathogens in the United States using disability adjusted life years. Epidemiol. Infect..

[bib219] Sears A., Baker M.G., Wilson N., Marshall J., Muellner P., Campbell D.M., Lake R.J., French N.P. (2011). Marked campylobacteriosis decline after interventions aimed at poultry, New Zealand. Emerg. Infect. Dis..

[bib220] Seguino A., Chintoan-Uta C., Smith S.H., Shaw D.J. (2018). Public health significance of *Campylobacter* spp. colonisation of wild game pheasants (*Phasianus colchicus*) in Scotland. Food Microbiol..

[bib221] Seliwiorstow T., Baré J., Verhaegen B., Uyttendaele M., De Zutter L. (2014). Evaluation of a new chromogenic medium for direct enumeration of Campylobacter in poultry meat samples. J. Food Prot..

[bib222] Seliwiorstow T., Baré J., Van Damme I., Uyttendaele M., De Zutter L. (2015). *Campylobacter* carcass contamination throughout the slaughter process of Campylobacter-positive broiler batches. Int. J. Food Microbiol..

[bib223] Sharma K.P., Chattopadhyay U.K., Naskar K. (2016). Prevalence of *Campylobacter* species in raw meat samples sold in open markets of Kolkata city. Int. J. Agric. Environ. Biotechnol..

[bib224] Schürch A.C., Arredondo-Alonso S., Willems R.J.L., Goering R.V. (2018). Whole genome sequencing options for bacterial strain typing and epidemiologic analysis based on single nucleotide polymorphism versus gene-by-gene–based approaches. Clin. Microbiol. Infect..

[bib225] Schulze F., H€anel I., Borrmann E. (1998). Formation of cytotoxins by enteric *Campylobacter* in humans and animals. Zentralblatt Bakteriol..

[bib226] Shane S.M. (2000). *Campylobacter* infection of commercial poultry. Revue Scientifique et Technique-Office Intern. des Epizoot..

[bib227] Shawky H.M., Kamel N.M., Farghaly E.M., Samir A. (2015). Isolation and molecular characterization of *Campylobacter* spp. in newly hatched poultry in Egypt. J. Global Biosci..

[bib228] Silva J., Leite D., Fernandes M., Mena C., Gibbs P.A., Teixeira P. (2011). *Campylobacter* spp. as a foodborne pathogen: a review. Front. Microbiol..

[bib229] Singhal N., Kumar M., Kanaujia P.K., Virdi J.S. (2015). MALDI-TOF mass spectrometry: an emerging technology for microbial identification and diagnosis. Front. Microbiol..

[bib230] Skarp C.P.A., Hänninen M.L., Rautelin H.I.K. (2016). Campylobacteriosis: the role of poultry meat. Clin. Microbiol. Infect..

[bib231] Skirrow M.B. (1977). Campylobacter enteritis: a “new” disease. Br. Med. J..

[bib232] Steens A., Eriksen H.M., Blystad H. (2014). What are the most important infectious diseases among those 65 years: a comprehensive analysis on notifiable diseases, Norway, 1993-2011. BMC Infect. Dis..

[bib233] Steinhauserova I., Fojtikova K., Klimes J. (2000). The incidence and PCR detection of *Campylobacter upsaliensis* in dogs and cats. Lett. Appl. Microbiol..

[bib234] Stuart T.L., Sandhu J., Stirling R., Corder J., Ellis A., Misa P., Goh S., Wong B., Martiquet P., Hoang L., Galanis E. (2010). Campylobacteriosis outbreak associated with ingestion of mud during a mountain bike race. Epidemiol. Infect..

[bib235] Sugrue I., Tobin C., Ross R.P., Stanton C., Hill C. (2019). Foodborne pathogens and zoonotic diseases. Raw Milk.

[bib236] Swierczewski B.E., Odundo E.A., Koech M.C., Ndonye J.N., Kirera R.K., Odhiambo C.P., Cheruiyot E.K., Shaffer D.N., Ombogo A.N., Oaks E.V. (2013). Enteric pathogen surveillance in a case-control study of acute diarrhea in Kisii Town, Kenya. J. Med. Microbiol..

[bib237] Szosland-Fałtyn A., Bartodziejska B., Królasik J., Paziak-Domańska B., Korsak D., Chmiela M. (2018). The prevalence of *Campylobacter* species in Polish poultry meat. Pol. J. Microbiol..

[bib238] Taheri N., Fällman M., Wai S.N., Fahlgren A. (2019). Accumulation of virulence-associated proteins in *Campylobacter jejuni* Outer Membrane Vesicles at human body temperature. J. Proteomics.

[bib239] Tam C.C., Rodrigues L.C., Viviani L., Dodds J.P., Evans M.R., Hunter P.R., Gray J.J., Letley L.H., Rait G., Tompkins D.S., O’Brien S.J. (2012). Longitudinal study of infectious intestinal disease in the UK (IID2 study): incidence in the community and presenting to general practice. Gut.

[bib240] Taylor P., D'Cruz P., Noronha E., Scholarios D. (2013). The experience of work in India's domestic call centre industry. Int. J. Hum. Resour. Manag..

[bib241] Thomas C.D. (2005). Pilot study for the development of a new *Campylobacter* selective medium at 37°C using aztreonam. J. Clin. Pathol..

[bib242] Thompson R.A., Monis P. (2012). Giardia from genome to proteome. Adv. Parasitol..

[bib243] Tribble D.R., Baqar S., Scott D.A., Oplinger M.L., Trespalacios F., Rollins D., Walker R.I., Clements J.D., Walz S., Gibbs P., Burg E.F.I., Moran A.P., Applebee L., Bourdeois A.L. (2010). Assessment of the duration of protection in *Campylobacter jejuni* experimental infection in humans. Infect. Immun..

[bib244] Unicomb L.E., Fullerton K.E., Kirk M.D., Stafford R.J. (2009). Outbreaks of campylobacteriosis in Australia, 2001 to 2006. Foodb. Pathog. Dis..

[bib245] Vacheyrou M., Normand A.C., Guyot P., Cassagne C., Piarroux R., Bouton Y. (2011). Cultivable microbial communities in raw cow milk and potential transfers from stables of sixteen French farms. Foodb. Pathog. Dis..

[bib246] van Vliet A.H.M., Ketley J.M. (2001). Pathogenesis of enteric *Campylobacter* infection. J. Appl. Microbiol..

[bib247] Vihavainen E., Lundstrom H.S., Susiluoto T., Koort J., Paulin L., Auvinen P., Bjorkroth J. (2007). Role of broiler carcasses and processing plant air in contamination of modified-atmosphere-packaged broiler products with psychrotrophic lactic acid bacteria. Appl. Environ. Microbiol..

[bib248] Wagenaar J.A., van Bergen M.A., Blaser M.J., Tauxe R.V., Newell D.G., van Putten J.P. (2014). *Campylobacter fetus* infections in humans: exposure and disease. Clin. Infect. Dis..

[bib249] Wang Y., Zhang M., Deng F., Shen Z., Wu C., Zhang J., Zhang Q., Shen J. (2014). Emergence of multidrug-resistant *Campylobacter* species isolates with a horizontally acquired rRNA methylase. Antimicrob. Agents Chemother..

[bib250] Walsh T.J., Roilides E., Rex J.H., McGinnis M.R. (2011). Mucormycosis. Trop. Med. Infect. Dis..

[bib251] Wei B., Kang M., Jang H.-K. (2018). Evaluation of potassium clavulanate supplementation of Bolton broth for enrichment and detection of *Campylobacter* from chicken. PLoS One.

[bib252] Wei B., Kang M. (2018). Molecular basis of macrolide resistance in *Campylobacter* strains isolated from poultry in South Korea. BioMed Res. Int..

[bib253] Weinberger M., Lerner L., Valinsky L., Moran-Gilad J., Nissan I., Agmon V., Peretz C. (2013). Increased incidence of *Campylobacter* spp. Infection and high rates among children. Israel Emerg. Infect. Dis..

[bib254] Werno A.M., Klena J.D., Shaw G.M., Murdoch D.R. (2002). Fatal case of *Campylobacter lari* prosthetic joint infection and bacteremia in an immunocompetent patient. J. Clin. Microbiol..

[bib255] Westermarck E. (2016). Chronic diarrhea in dogs: what do we actually know about it?. Top. Companion Anim. Med..

[bib256] WHO (2013). The Global View of Campylobacteriosis: Report of an Expert Consultation. Utrecht, Netherlands 9-11 July 2012. Who Rep. 57.

[bib257] WHO (2018). WHO Estimates of the Global Burden of Foodborne Diseases: Foodborne Disease Burden Epidemiology Reference Group 2007-2015.

[bib258] Wieczorek K., Osek J. (2013). Antimicrobial resistance mechanisms among *Campylobacter*. BioMed Res. Int..

[bib259] Wieczorek K., Denis E., Osek J. (2015). Comparative analysis of antimicrobial resistance and genetic diversity of *Campylobacter* from broilers slaughtered in Poland. Int. J. Food Microbiol..

[bib260] Wieczorek K.A., Wołkowicz T., Osek J. (2018). Antimicrobial resistance and virulence-associated traits of *Campylobacter jejuni* isolated from poultry food chain and humans with diarrhea. Front. Microbiol..

[bib261] Wilkinson D.A., O’Donnell A.J., Akhter R.N., Fayaz A., Mack H.J., Rogers L.E., Biggs P.J., French N.P., Midwinter A.C. (2018). Updating the genomic taxonomy and epidemiology of *Campylobacter hyointestinalis*. Sci. Rep..

[bib262] Wilson D.J., Gabriel E., Leatherbarrow A.J., Cheesbrough J., Gee S., Bolton E., Fox A., Fearnhead P., Hart C.A., Diggle P.J. (2008). Tracing the source of campylobacteriosis. PLoS Genet..

[bib263] Wong T.L., Hollis L., Cornelius A., Nicol C., Cook R., Hudson J.A. (2007). Prevalence, numbers, and subtypes of *Campylobacter jejuni* and *Campylobacter coli* in uncooked retail meat samples. J. Food Prot..

[bib264] Wood T.K., Knabel S.J., Kwan B.W. (2013). Bacterial persister cell formation and dormancy. Appl. Environ. Microbiol..

[bib265] Zaidi M.B., Campos F.D., Estrada-Garcia T., Gutierrez F., Leon M., Chim R., Calva J.J. (2012). Burden and transmission of zoonotic foodborne disease in a rural community in Mexico. Clin. Infect. Dis..

